# The use of different artificial intelligence-powered chatbots to answer questions related to dental implants

**DOI:** 10.3389/froh.2026.1975960

**Published:** 2026-10-21

**Authors:** Rasha A. Alamoush, Mohammad Atrash, Ziad Abusidu, Alaa H. A. Sabrah, Islam Abd Alraheam, Marwa M. Alnsour, Mahmoud K. AL-Omiri

**Affiliations:** 1 Department of Fixed and Removable Prosthodontics, School of Dentistry, The University of Jordan, Amman, Jordan; 2 Department of Dentistry, Jordan University Hospital, Amman, Jordan; 3 Department of Dentistry, Komar University of Science and Technology, Sulaymaniyah, Iraq; 4 Department of Restorative Dentistry, School of Dentistry, University of Jordan, Amman, Jordan

**Keywords:** artificial intelligence, chatbots, dental implants, digital dentistry, health literacy, large language models, patient education

## Abstract

**Background:**

Artificial intelligence (AI)-powered chatbots based on large language models (LLMs) are increasingly used as sources of health information. In dentistry, patients frequently seek online information regarding dental implant therapy; however, the quality, reliability, and clinical usefulness of AI-generated responses remain uncertain. This study aimed to compare the performance of commonly available AI chatbots in answering dental implant-related questions.

**Methods:**

Ninety-nine publicly available dental implant-related questions were identified using online question-generation platforms and categorized into four domains: biological and surgical aspects, prosthetic and material science, clinical outcomes and complications, and patient-centered and economic considerations. Each question was submitted independently to four AI chatbots: ChatGPT-5, Gemini 3 Pro, DeepSeek V3.2, and Microsoft Copilot. Responses were evaluated by two calibrated dental specialists using the Global Quality Scale (GQS), CLEAR reliability assessment tool, usefulness scoring, and readability indices including Flesch–Kincaid Grade Level (FKG) and Simple Measure of Gobbledygook (SMOG). Statistical analyses were performed using appropriate parametric and non-parametric tests with a significance level of *p* < 0.05.

**Results:**

All evaluated chatbots generated responses with very good reliability according to CLEAR scores. Significant differences were observed among AI models in readability, quality, usefulness, and reliability scores (*p* < 0.001). DeepSeek demonstrated the highest reliability (median CLEAR score = 25) and the highest proportion of excellent-quality responses according to GQS. ChatGPT generated significantly more useful responses compared with other models. DeepSeek and Copilot produced more readable responses, whereas Gemini demonstrated the highest reading difficulty based on FKG and SMOG scores. Response performance was not significantly influenced by the question category.

**Conclusions:**

AI-powered chatbots can provide generally reliable information regarding dental implants and may serve as supportive sources of patient education. However, differences exist between AI models regarding readability, usefulness, and response quality. AI-generated information should complement rather than replace professional dental consultation, and further studies involving patients and clinically validated outcomes are warranted.

## Introduction

The rapid advancement of artificial intelligence (AI) and digital technologies has transformed the way individuals seek medical and dental information. Increasingly, patients rely on the internet and digital health resources to obtain information about diseases, treatment options, and healthcare services before consulting healthcare professionals (1–8). Although conventional online resources, such as search engines and health-related websites, provide extensive information, users are often required to review multiple sources, assess their credibility, and interpret complex medical content, which may be challenging and time-consuming (3–8).

Recent developments in AI have introduced large language models (LLMs), a significant advance in digital health communication. Unlike traditional search engines or static educational materials, LLMs generate human-like responses through natural language processing and extensive training on large text-based datasets, enabling them to provide contextually relevant, conversational answers to user queries (6, 9). Among the most widely used LLMs are ChatGPT, developed by OpenAI, and Gemini, developed by Google. These AI-powered chatbots have demonstrated considerable potential in healthcare communication by generating personalised, accessible, and contextually appropriate responses using advanced natural language processing techniques (4, 5, 10). ChatGPT has been recognised for its conversational fluency and broad knowledge base (11, 12), whereas Gemini has shown promising performance in medical-domain understanding and reasoning (13, 14). Consequently, these models have the potential to improve patient engagement, support health literacy, and facilitate access to medical information across various healthcare disciplines, including dentistry (1, 15, 16). Furthermore, these models can adapt their responses based on user interactions, clarify misconceptions, and engage in dynamic conversations, thereby enhancing the accessibility and personalisation of health information (1, 11, 12).

Dental implant therapy is a predictable and evidence-based treatment for replacing missing teeth and has become one of the most commonly performed restorative procedures worldwide. Despite its high success rates and increasing popularity, many patients have limited knowledge regarding implant treatment. Common areas of uncertainty include treatment planning, surgical and prosthetic procedures, available treatment options, longevity, maintenance requirements, costs, and potential complications (17, 19). Consequently, patients frequently seek information from online resources before or after consulting their dentists.

Patient education is fundamental to obtaining informed consent, reducing treatment-related anxiety, improving adherence to postoperative instructions, and ultimately enhancing clinical outcomes. Traditionally, patient education has relied on face-to-face consultations, printed educational materials, and multimedia resources. However, these approaches may be limited by time constraints, accessibility, and variability in the quality and consistency of the information provided (19). AI-powered chatbots offer an alternative approach by providing immediate, personalized, and interactive responses to patients' questions, potentially complementing conventional patient education methods.

Although LLMs have demonstrated promising performance across several medical and dental applications, concerns remain regarding the accuracy, reliability, completeness, and clinical applicability of the information they generate (20, 21). Previous studies evaluating AI chatbots in dentistry have generally reported that these systems provide useful and understandable information; however, inaccuracies, incomplete responses, and limited actionable guidance have also been identified, highlighting the need for careful evaluation before their integration into routine patient education (18–24).

Dental implant therapy represents one of the most common topics for which patients seek online information because of its complexity and the wide range of available treatment options. Therefore, assessing the quality and reliability of AI-generated responses to publicly available dental implant-related questions is important to determine whether these tools can safely support patient education and supplement professional consultation.

To the best of the authors' knowledge, few studies have comprehensively compared multiple AI-powered chatbots in answering patient questions related to dental implants. Therefore, this study aimed to evaluate the performance of commonly available AI-powered chatbots in responding to publicly available questions about dental implants. Specifically, the study assessed the quality, reliability, and clinical applicability of chatbot-generated responses across key domains of dental implant therapy.

## Methodology

### Question identification and selection

To identify commonly asked public questions regarding dental implants, an online exploratory search was conducted using three widely used question-suggestion tools: “Also Asked” (https://alsoasked.com/), “Answer the Public” (https://answerthepublic.com/), and Google's “People Also Ask” feature. Searches were performed using relevant keywords, including *dental implants, implant pain, implant complications, implant cost, implant longevity,* and related variations.

A total of 176 questions were initially generated. Duplicate questions and semantically identical items were removed independently by two reviewers. Any disagreements were resolved through discussion, and when necessary, consultation with a third senior reviewer. After refinement, 99 unique questions remained. All questions were reviewed for grammatical accuracy and clarity prior to inclusion. The final set was categorized into four domains:

1. Biological and surgical aspects
2. Prosthetic and material science
3. Clinical outcomes and complications
4. Patient-centered and economic considerations

The questions were assigned to the four domains according to their primary subject matter. Therefore, the number of questions in each domain was not predetermined or equalized, but reflected the topics represented among the identified questions.

### AI chatbot response generation

The finalized questions were submitted to four AI platforms: ChatGPT-5 (OpenAI, San Francisco, CA, USA), Gemini 3 Pro (Google, San Francisco, CA, USA), DeepSeek V3.2 (DeepSeek, Hangzhou, China), and Microsoft Copilot (Microsoft Corporation). Data were collected on December 4, 2025, using the free consumer versions of all platforms without paid subscriptions. To ensure consistency and minimize potential bias, all platforms were accessed through incognito browser windows, with no search history or retrieval tools available. Each question was entered individually in a new conversation, and the webpage was refreshed before entering each subsequent question to prevent contextual carryover between responses. The “regenerate response” option was not used, and the default settings of each platform were retained. Responses were independently collected by one investigator and stored in anonymized Microsoft Word documents to facilitate blinded evaluation by the two assessors, with the assigned scores concealed from the evaluators.

### AI response evaluation

The evaluation of AI responses was structured across four primary domains:

1. Quality of responses—assessed using the Global Quality Scale (GQS), which evaluates overall response quality, coherence, flow, and relevance to patient needs. Higher scores indicate more comprehensive and clinically appropriate responses.
2. Reliability—assessed using the CLEAR framework developed by Sallam et al., which evaluates five domains (25): completeness, lack of false information, evidence support, appropriateness, and relevance. Each domain was scored on a five-point scale, ranging from 1 (poor) to 5 (excellent), resulting in a total score ranging from 5 to 25, with higher scores indicating greater reliability and quality. For descriptive interpretation, total CLEAR scores of 5–11, 12–18, and 19–25 were classified as poor, average, and very good quality, respectively.
3. Usefulness—assessed using a modified 4-point Likert scale, defined as:
  1 = Very useful (provides comprehensive, accurate information comparable to specialist level), 2 = Useful (generally accurate but missing minor or non-critical details), 3 = Partially useful (contains a mixture of correct and incorrect or incomplete information), 4 = Not useful (contains inaccurate, misleading, or clinically inappropriate information).

Readability—assessed using the Flesch–Kincaid Reading Ease/Grade Level (FKG) and the Simple Measure of Gobbledygook (SMOG) index. The FKG estimates the U.S. school grade level required to understand the text as it gives a marker of readability with a weighting on syllables. It can be calculated with the following formula: 0.39 (total words/total sentences) + 11.8 (total syllables/total words)– 15.59. the result estimates the educational United States grade level equivalent. when a text has an FKG score of 8, the reader needs a grade level of 8 to read and understand its meaning (26). while the SMOG index which created by a clinical psychologist (G Harry McLaughlin) estimates the number of years of formal education that a person needs to read the material. The SMOG formula is: 3+ square root √ [number of polysyllabic words×(30 ÷ number of sentences)]. It is widely used, especially in the healthcare sector, A score under 9 is suggestive of an easy-to-read text (education level required: middle school) (27).

Together, these indices provide an objective assessment of the accessibility of the generated responses.

This structured multi-domain assessment framework was used to ensure a comprehensive evaluation of AI-generated responses in terms of quality, reliability, usefulness, and readability, thereby supporting robust comparative analysis across evaluators. It is worth noting that a response being correct and reliable from the evaluators' point of view does not necessarily imply it would be beneficial to patients. Both CLEAR and GQS measure the quality of the response and the trustworthiness of the information. On the other hand, Usefulness measures how much a response is useful for patient needs.

### Test-retest reliability assessment

AI-generated responses were independently evaluated by two board-certified specialists in fixed prosthodontics who were trained in the use of standardized assessment instruments. Prior to the main evaluation, a calibration phase was conducted using a pilot dataset of 40 question–answer pairs that were excluded from the final analysis (initially generated question were 176, only 99 questions were involved in the final analysis). In the calibration phase, the AI-generated responses were independently evaluated by the same board-certified specialists in fixed prosthodontics. This process was designed to ensure consistency in interpretation and application of the evaluation criteria across raters. Any discrepancies in scoring during this phase were discussed, and unresolved disagreements were adjudicated by a third senior expert in fixed prosthodontics to establish consensus and standardize scoring procedures.

The inter-rater reliability for evaluation of GQS, usefulness, and CLEAR total and individual element scores was evaluated by comparing the two evaluators assessment of the values of responses. Kappa values were 0.787, 0.838, 0.750, 0.737, 0.808, 0.846, and 0.741 for GQS scores, usefulness scores, completeness, lack of false information, evidence, appropriateness, and relevance CLEAR scores, respectively. For total CLEAR score values, the intra-class correlation coefficient value was 0.876 (95% CI: 0.760–0.935). Therefore, the inter-examiner test-retest reliability was adequate and indicated proper standardized assessment process by both investigators in this investigation.

### Statistical analysis

Data analysis was performed utilizing the SPSS computer software (IBM SPSS Statistics v20.0; IBM Corp., USA). Two-tailed *α* at 0.05% and 95% confidence intervals were considered to identify significant statistical outcomes. Shapiro–Wilk test, Q-Q plots, and skewness test (skewness z scores of variables between −3.29 and +3.29) were used to test normal distribution of the data. FKG and SMOG scores were normally distributed while total CLEAR scores were not normally distributed. Therefore, means, standard errors, standard deviations, 95% confidence intervals, median, maximum values, and minimum values were used to describe FKG and SMOG scores. Meanwhile, medians, interquartile ranges, minimum values, and maximum values were used to describe CLEAR total and individual element scores. Also, frequencies and percentages were utilized to report the categorical data (CLEAR individual elements, usefulness, and GQS scores).

Factorial mixed design ANOVA models were used to test variations in FKG and SMOG scores in view of the main and interactions effects of the type of artificial intelligence chatbot and the category of generated questions. Pairwise comparisons (with multiple comparison correction) were then conducted utilizing the Bonferroni *post hoc* test.

Variations in CLEAR total, usefulness, and GQS scores between different artificial intelligence powered chatbots were tested via Friedman's test followed by Wilcoxon-Signed ranks test for pairwise comparisons. Variations in CLEAR total, usefulness, and GQS scores between categories of questions regardless the AI chatbot were tested using the Kruskal Wallis test followed by Mann Whitney U test for pairwise comparisons. The Benjamini-Hochberg method was used for correction to multiple pairwise comparisons obtained via Wilcoxon Signed Ranks test and Mann Whitney U test. It was utilized to adjust for the multiple comparisons and testing by controlling the false discovery rate (FDR) at 5%.

Correlations between normally distributed variables (FKG and SMOG scores) were tested via Pearson correlation test. Meanwhile, correlations involved CLEAR total, usefulness, and GQS scores (not normally distributed) were tested via Spearman's Rho correlation test.

## Results

### Search output

For this investigation, 99 questions were generated and analyzed. The generated questions were classified into 4 categories including biological and surgical aspects (*n* = 16 questions, % = 16.2), prosthetic and material science (*n* = 22 questions, % = 22.2), clinical outcomes and complications (*n* = 26 questions, % = 26.3), and patient centered and economic factors (*n* = 35 questions, % = 35.4).

Table 1 shows the descriptive statistics of FKG, SMOG, CLEAR total and individual element, GQS, and usefulness scores based on AI chatbot and category of questions. Figure 1 demonstrates the distribution of FKG, SMOG, and CLEAR total scores based on AI chatbot and category of questions. Supplementary Table S1 presents the descriptive statistics of CLEAR individual element, GQS, and usefulness scores based on AI chatbot and category of questions. Supplementary Table S2 presents the frequencies and percentages of each scale answer for CLEAR individual elements based on AI chatbot and category of questions. Figure 2 presents the distribution of CLEAR element scores based on AI chatbot and category of questions.

**Table 1:** Distribution of FKG, SMOG, CLEAR total and individual element, GQS, and usefulness scores based on AI chatbot and category of questions in this study (*n* = 99 questions).

Variable	Statistic	AI Chatbot	^a^Question Category				
			All (*n* = 99)	1 (*n* = 16)	2 (*n* = 22)	3 (*n* = 26)	4 (*n* = 35)
Flesch-Kincaid Grade Level readability score	Mean	ChatGPT	9.42	9.99	9.15	9.85	9.00
	Standard deviation		2.23	1.89	2.38	2.08	2.34
	Standard error		0.22	0.47	0.51	0.41	0.40
	Median		9.40	9.75	9.65	9.45	8.40
	Minimum value		4.10	7.10	5.10	6.00	4.10
	Maximum value		15.30	12.70	12.80	15.30	14.00
	Lower 95% CI		8.97	8.98	8.10	9.01	8.20
	Upper 95% CI		9.86	10.99	10.21	10.70	9.81
	Mean	Gemini	11.07	11.35	10.81	10.80	11.31
	Standard deviation		1.87	1.73	1.59	1.42	2.35
	Standard error		0.19	0.43	0.34	0.28	0.40
	Median		10.90	11.40	10.85	10.80	10.90
	Minimum value		6.10	8.20	7.00	8.00	6.10
	Maximum value		15.50	14.20	14.40	13.50	15.50
	Lower 95% CI		10.70	10.43	10.11	10.22	10.50
	Upper 95% CI		11.44	12.27	11.52	11.37	12.12
	Mean	DeepSeek	8.67	8.53	8.60	8.85	8.63
	Standard deviation		1.06	1.05	1.00	1.10	1.11
	Standard error		0.11	0.26	0.21	0.22	0.19
	Median		8.60	8.45	8.60	8.80	8.50
	Minimum value		6.30	7.00	7.00	6.30	6.30
	Maximum value		11.10	10.80	10.90	11.10	10.90
	Lower 95% CI		8.45	7.97	8.15	8.41	8.25
	Upper 95% CI		8.88	9.09	9.04	9.30	9.01
	Mean	Co-Pilot	9.02	8.89	8.75	9.38	8.97
	Standard deviation		1.36	1.37	1.31	1.30	1.43
	Standard error		0.14	0.34	0.28	0.25	0.24
	Median		8.80	8.60	8.60	9.35	8.70
	Minimum value		6.60	6.60	7.00	7.30	7.20
	Maximum value		12.60	12.10	11.60	11.90	12.60
	Lower 95% CI		8.74	8.16	8.17	8.86	8.48
	Upper 95% CI		9.29	9.63	9.32	9.91	9.46
SMOG readability score	Mean	ChatGPT	11.64	12.11	11.20	11.94	11.48
	Standard deviation		1.76	1.53	1.51	1.65	2.03
	Standard error		0.18	0.38	0.32	0.32	0.34
	Median		11.50	12.10	11.55	11.55	11.20
	Minimum value		7.90	9.30	8.70	9.20	7.90
	Maximum value		16.50	14.20	14.10	16.30	16.50
	Lower 95% CI		11.29	11.30	10.53	11.27	10.78
	Upper 95% CI		11.99	12.93	11.87	12.60	12.18
	Mean	Gemini	13.18	13.34	13.06	12.92	13.37
	Standard deviation		1.44	1.23	1.19	1.23	1.80
	Standard error		0.15	0.31	0.25	0.24	0.30
	Median		13.10	13.05	13.20	13.00	13.30
	Minimum value		9.10	11.00	10.00	10.60	9.10
	Maximum value		16.70	15.30	15.70	15.50	16.70
	Lower 95% CI		12.89	12.68	12.53	12.43	12.75
	Upper 95% CI		13.46	13.99	13.58	13.42	13.98
	Mean	DeepSeek	11.39	11.22	11.42	11.44	11.42
	Standard deviation		0.85	0.83	0.75	0.99	0.84
	Standard error		0.09	0.21	0.16	0.19	0.14
	Median		11.40	10.95	11.55	11.35	11.40
	Minimum value		9.40	10.10	10.00	9.40	9.70
	Maximum value		13.50	13.00	13.00	13.50	13.30
	Lower 95% CI		11.22	10.78	11.09	11.04	11.13
	Upper 95% CI		11.56	11.66	11.76	11.83	11.71
	Mean	Co-Pilot	11.11	11.00	11.07	11.30	11.04
	Standard deviation		1.02	1.01	1.03	1.02	1.05
	Standard error		0.10	0.25	0.22	0.20	0.18
	Median		11.00	10.90	11.00	11.30	10.90
	Minimum value		9.40	9.40	9.50	9.60	9.50
	Maximum value		14.00	13.50	12.80	13.00	14.00
	Lower 95% CI		10.90	10.46	10.62	10.89	10.68
	Upper 95% CI		11.31	11.54	11.53	11.71	11.40
CLEAR reliability total score	Mean	ChatGPT	23.31	23.00	23.09	23.15	23.71
	Standard deviation		1.234	1.673	1.151	1.047	1.126
	Median		24.00	22.50	23.00	23.00	24.00
	Minimum value		19	19	20	20	19
	Maximum value		25	25	25	24	25
	Inter quartile range		1	3	2	1	1
	Mean	Gemini	23.44	23.63	23.00	23.54	23.57
	Standard deviation		1.401	1.500	1.512	1.363	1.313
	Median		24.00	24.00	23.00	24.00	24.00
	Minimum value		19	20	20	19	19
	Maximum value		25	25	25	25	25
	Inter quartile range		1	2	2	1	1
	Mean	DeepSeek	24.68	24.69	24.36	24.81	24.77
	Standard deviation		.712	.479	1.217	.491	.426
	Median		25.00	25.00	25.00	25.00	25.00
	Minimum value		20	24	20	23	24
	Maximum value		25	25	25	25	25
	Inter quartile range		0	1	1	0	0
	Mean	Co-Pilot	23.65	23.50	23.32	23.54	24.00
	Standard deviation		1.043	.730	1.673	.706	.767
	Median		24.00	23.50	24.00	24.00	24.00
	Minimum value		19	22	19	22	23
	Maximum value		25	25	25	25	25
	Inter quartile range		1	1	1	1	2

a Question categories: 1, Biological and surgical aspects; 2, Prosthetic and material science; 3, Clinical outcomes and complications; and 4, Patient centered and economic factors. SMOG, Simple Measure of Gobbledygook readability index; CLEAR, Completeness; Lack of false information; Evidence support; Appropriateness; and Relevance reliability tool; CI, Confidence intervals.

**Figure 1:**
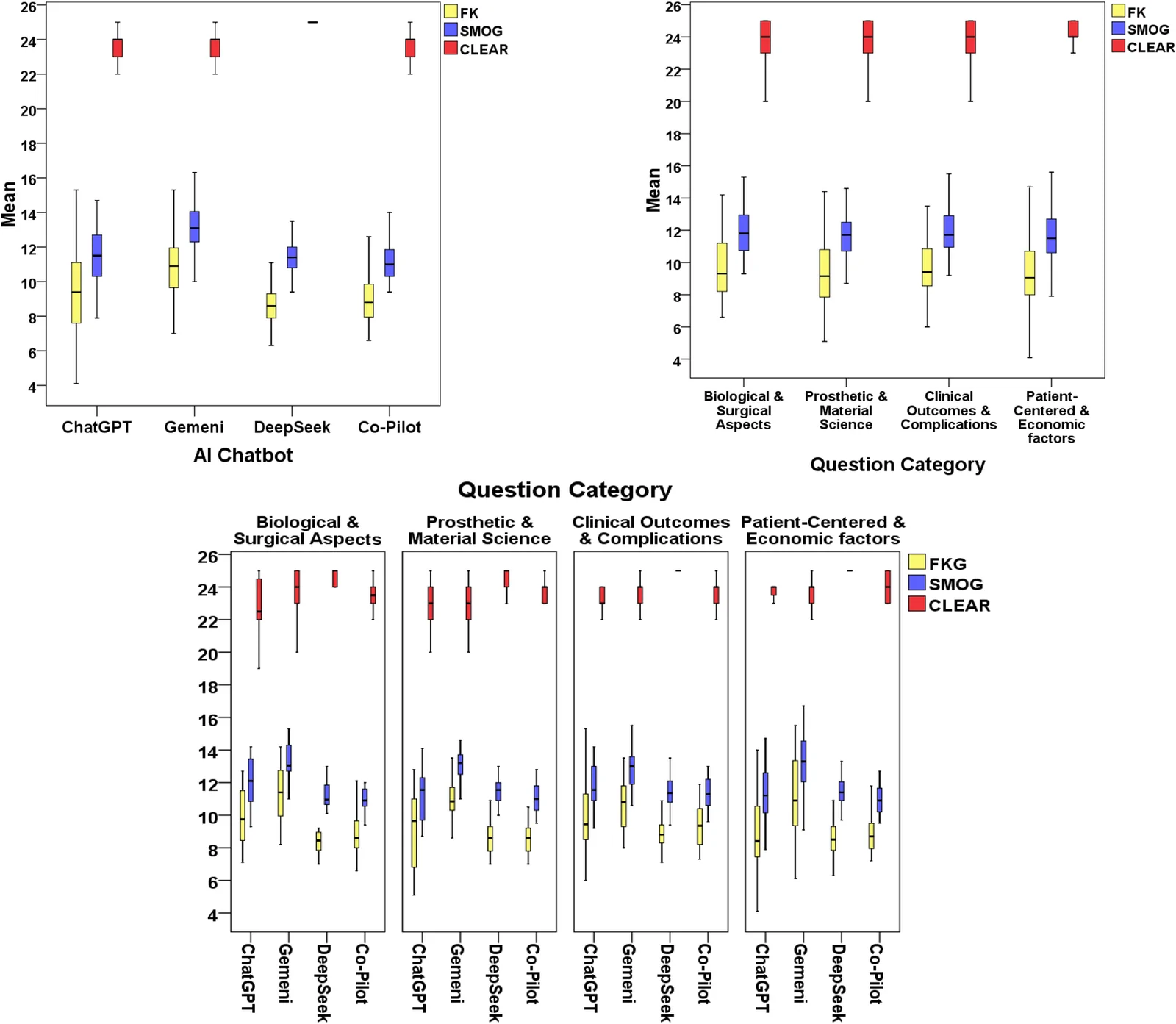
Boxplots of the distribution of FKG, SMOG, and CLEAR total scores based on AI chatbot and category of questions (*n* = 99).

**Figure 2:**
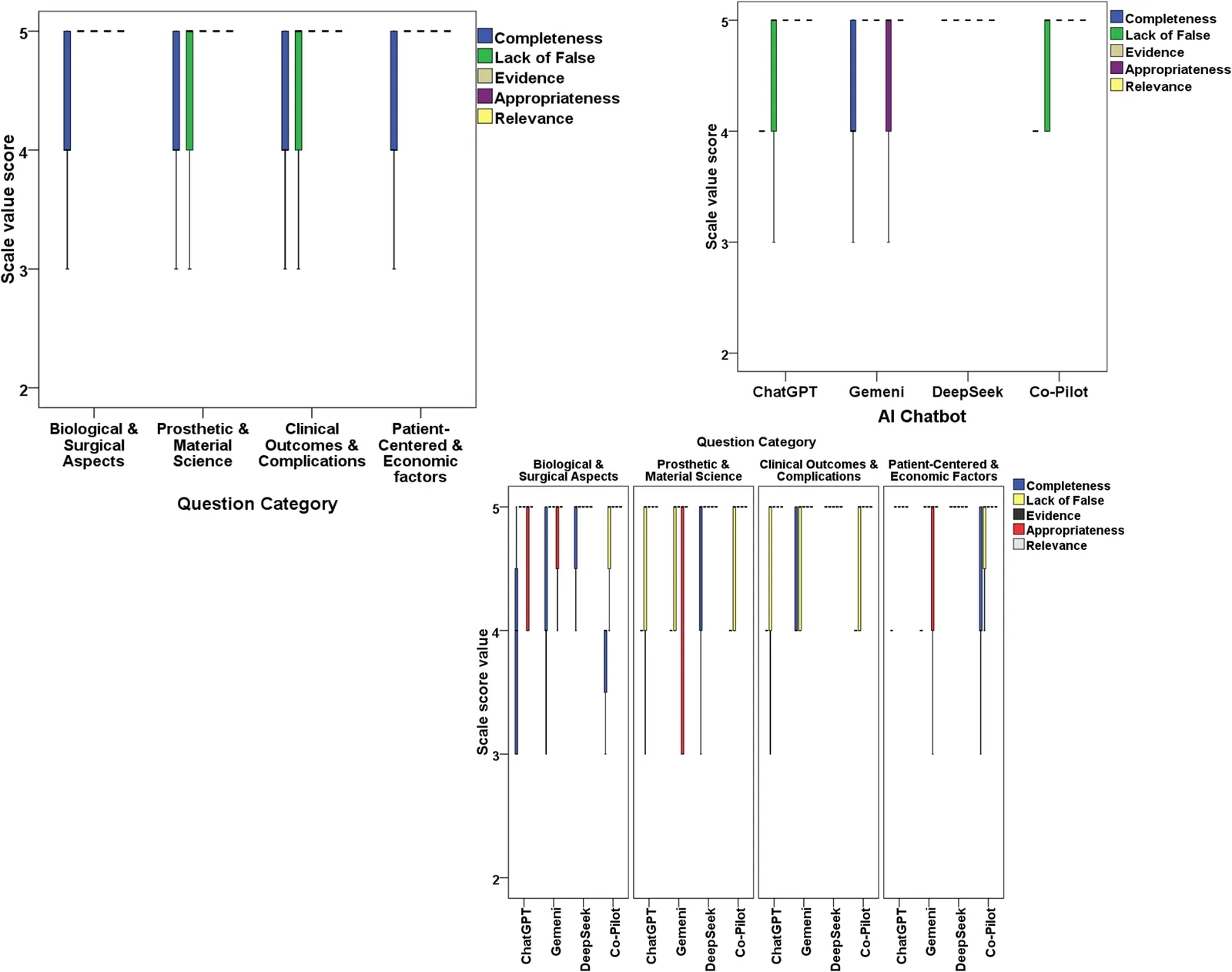
Boxplots of the distribution of CLEAR element scores based on AI chatbot and category of questions (*n* = 99).

Table 2 presents frequencies and percentages of interpretations and categorizations of scale scores and answers for SMOG, CLEAR total, GQS, and usefulness scores based on AI chatbot and category of questions in this study.

**Table 2:** Frequencies and percentages of interpretations and categorizations of scale scores and answers for SMOG, CLEAR total, GQS, and usefulness scores based on AI chatbot and category of questions in this study (*n* = 99 questions).

Variable	Interpretation/ answer category	AI Chatbot	^a^Question Category									
			All (*n* = 99)		1 (*n* = 16)		2 (*n* = 22)		3 (*n* = 26)		4 (*n* = 35)	
			*n*	%	*n*	%	*n*	%	*n*	%	*n*	%
SMOG score 6–7 = Fairly easy, 8–9 = Moderately easy, 10–12 = Moderately difficult, >13 = Challenging to read.	Fairly easy	ChatGPT	0	0	0	0	0	0	0	0	0	0
	Moderately easy		10	10.1	1	6.3	4	18.2	1	3.8	4	11.4
	Moderately difficult		59	59.6	8	50.0	14	63.6	16	61.6	21	60.0
	Challenging		30	30.3	7	43.7	4	18.2	9	34.6	10	28.6
	Fairly easy	Gemini	0	0	0	0	0	0	0	0	0	0
	Moderately easy		1	1	0	0	0	0	0	0	1	2.9
	Moderately difficult		27	27.3	3	18.8	5	22.7	8	30.8	11	31.4
	Challenging		71	71.7	13	81.2	17	77.3	18	69.2	23	65.7
	Fairly easy	DeepSeek	0	0	0	0	0	0	0	0	0	0
	Moderately easy		1	1	0	0	0	0	1	3.8	0	0
	Moderately difficult		88	88.9	14	87.5	21	95.5	22	84.7	31	88.6
	Challenging		10	10.1	2	12.5	1	4.5	3	11.5	4	11.4
	Fairly easy	Co-Pilot	0	0	0	0	0	0	0	0	0	0
	Moderately easy		1	1	1	6.3	0	0	0	0	0	0
	Moderately difficult		85	85.9	14	87.5	19	86.4	21	80.8	31	88.6
	Challenging		13	13.1	1	6.3	3	13.6	5	19.2	4	11.4
CLEAR Total score 5–11 = Poor content, 12–18 = Average content, 19–25 = Very good content	Poor content	ChatGPT	0	0	0	0	0	0	0	0	0	0
	Average		0	0	0	0	0	0	0	0	0	0
	Very good		99	100.0	16	100.0	22	100.0	26	100.0	35	100.0
	Poor content	Gemini	0	0	0	0	0	0	0	0	0	0
	Average		0	0	0	0	0	0	0	0	0	0
	Very good		99	100.0	16	100.0	22	100.0	26	100.0	35	100.0
	Poor content	DeepSeek	0	0	0	0	0	0	0	0	0	0
	Average		0	0	0	0	0	0	0	0	0	0
	Very good		99	100.0	16	100.0	22	100.0	26	100.0	35	100.0
	Poor content	Co-Pilot	0	0	0	0	0	0	0	0	0	0
	Average		0	0	0	0	0	0	0	0	0	0
	Very good		99	100.0	16	100.0	22	100.0	26	100.0	35	100.0
Usefulness score Very useful = 1, Useful = 2, Partially useful = 3, Not useful = 4	Very useful	ChatGPT	1	1.0	0	0	0	0	1	3.8	0	0
	Useful		42	42.4	7	43.8	10	45.5	10	38.5	15	42.9
	Partially useful		51	51.5	7	43.8	12	54.5	15	57.7	17	48.6
	Not useful		5	5.1	2	12.5	0	0	0	0	3	8.6
	Very useful	Gemini	0	0	0	0	0	0	0	0	0	0
	Useful		27	27.3	3	18.8	7	31.8	9	34.6	8	22.9
	Partially useful		66	66.7	12	75.0	13	59.1	16	61.5	25	71.4
	Not useful		6	6.1	1	6.3	2	9.1	1	3.8	2	5.7
	Very useful	DeepSeek	0	0	0	0	0	0	0	0	0	0
	Useful		1	1.0	0	0	0	0	1	3.8	0	0
	Partially useful		59	59.6	10	62.5	16	72.7	12	46.2	21	60.0
	Not useful		39	39.4	6	37.5	6	27.3	13	50.0	14	40.0
	Very useful	Co-Pilot	0	0	0	0	0	0	0	0	0	0
	Useful		27	27.3	7	43.8	4	18.2	9	34.6	7	20.0
	Partially useful		67	67.7	9	56.3	16	72.7	16	61.5	26	74.3
	Not useful		5	5.1	0	0	2	9.1	1	3.8	2	5.7
GQS score Poor quality = 1, Generally poor = 2, Moderate = 3, Good = 4, Excellent = 5	Poor quality	ChatGPT	0	0	0	0	0	0	0	0	0	0
	Generally poor		2	2.0	0	0	1	4.5	1	3.8	0	0
	Moderate		13	13.1	1	6.3	2	9.1	8	30.8	2	5.7
	Good		70	70.7	11	68.8	16	72.7	16	61.5	27	77.1
	Excellent		14	14.1	4	25.0	3	13.6	1	3.8	6	17.1
	Poor quality	Gemini	0	0	0	0	0	0	0	0	0	0
	Generally poor		0	0	0	0	0	0	0	0	0	0
	Moderate		5	5.1	1	6.3	1	4.5	1	3.8	2	5.7
	Good		70	70.7	10	62.5	15	68.2	18	69.2	27	77.1
	Excellent		24	24.2	5	31.3	6	27.3	7	26.9	6	17.1
	Poor quality	DeepSeek	0	0	0	0	0	0	0	0	0	0
	Generally poor		0	0	0	0	0	0	0	0	0	0
	Moderate		0	0	0	0	0	0	0	0	0	0
	Good		16	16.2	4	25.0	7	31.8	1	3.8	4	11.4
	Excellent		83	83.8	12	75.0	15	68.2	25	96.2	31	88.6
	Poor quality	Co-Pilot	0	0	0	0	0	0	0	0	0	0
	Generally poor		0	0	0	0	0	0	0	0	0	0
	Moderate		7	7.1	0	0	3	13.6	3	11.5	1	2.9
	Good		82	82.8	15	93.8	14	63.6	23	88.5	30	85.7
	Excellent		10	10.1	1	6.3	5	22.7	0	0	4	11.4

a Question categories: 1, Biological and surgical aspects; 2, Prosthetic and material science; 3, Clinical outcomes and complications; 4, Patient centered and economic factors. SMOG, Simple Measure of Gobbledygook readability index; CLEAR, Completeness; Lack of false information; Evidence support; Appropriateness; and Relevance reliability tool; GQS, Global Quality Scale.

### Readability of AI chatbot generated responses

Considering all questions, DeepSeek AI chatbot (mean ± SD = 8.67 ± 1.06) was associated with the lowest mean FKG scores (i.e., the easiest to read answers); followed by Co-Pilot (mean ± SD = 9.02 ± 1.36), ChatGPT (mean ± SD = 9.42 ± 2.23), and Gemini (mean ± SD = 11.07 ± 1.87) in an increasing order of difficulty to read answers (Table 1). Similar trends were also found within each question category as DeepSeek AI chatbot was associated with the lowest mean FKG scores (i.e., the easiest to read answers) for biological and surgical aspects question category (mean ± SD = 8.53 ± 1.05), prosthetic and material science (mean ± SD = 8.60 ± 1.00), clinical outcomes and complications (mean ± SD = 8.85 ± 1.10), and patient centered and economic factors (mean ± SD = 8.63 ± 1.11); followed by Co-Pilot, ChatGPT, and Gemini in an increasing order of difficulty to read answers in each question category (Table 1 and Figure 1).

Considering all questions, Co-Pilot AI chatbot (mean ± SD = 11.11 ± 1.02) was associated with the lowest mean SMOG scores (i.e., the easiest to read answers); followed by DeepSeek (mean ± SD = 11.39 ± 0.85), ChatGPT (mean ± SD = 11.64 ± 1.76), and Gemini (mean ± SD = 13.18 ± 1.44) in an increasing order of difficulty to read answers (Table 1). Similar trends were also found within each question category as Co-Pilot AI chatbot was associated with the lowest mean SMOG scores (i.e., the easiest to read answers) for biological and surgical aspects question category (mean ± SD = 11.00 ± 1.01), prosthetic and material science (mean ± SD = 11.07 ± 1.03), clinical outcomes and complications (mean ± SD = 11.30 ± 1.02), and patient centered and economic factors (mean ± SD = 11.04 ± 1.05); followed by DeepSeek, ChatGPT, and Gemini in an increasing order of difficulty to read answers in each question category (Table 1 and Figure 1). Based on SMOG scores, most answers were classified and interpreted as moderately difficult to read for each AI chatbot considering all questions as well as for each question category, except for Gemini AI chatbot (Table 2). Gemini AI chatbot was mainly associated with challenging to read responses considering all questions (71.1%) as well as for each question category including biological and surgical aspects (81.2%), prosthetic and material science (77.3%), clinical outcomes and complications (69.2%), and patient centered and economic factors (65.7%) question categories (Table 2).

A mixed design ANOVA test (Table 3) showed significant differences in FKG scores between the tested AI chatbots in this study (main effects: F(2.531) = 60.695, *P* < .001, effect size *η*^2^ = 0.390). This indicated that the choice of AI chatbot strongly and reliably explains a large portion of the differences in FKG scores (39% of the differences). Further pairwise comparisons showed that Gemini AI chatbot was associated with higher FKG scores (i.e., more difficult to read answers) than ChatGPT, DeepSeek, and Co-Pilot AI chatbots (*P* < .001, Table 3). Also, ChatGPT AI chatbot had higher FKG scores (i.e., more difficult to read answers) than DeepSeek AI chatbot (*P* < .001, Table 3).

**Table 3:** A mixed design ANOVA test of main and interaction effects of different AI chatbots and question categories on variations in FKG and SMOG scores in this study (*n* = 99 questions).

Independent variable	Factor	Mixed design ANOVA	Bonferroni *post hoc* test between pairs					
			Compared pairs	MD	SE	*P*	CI	ES
FKG scores	AI Chatbot (main effects)	*F* = 60.695	ChatGPT vs. Gemini	−1.569	.238	<.001	−2.209––.928	0.80
		df = 2.531^b^	ChatGP vs. DeepSeek	.846	.197	<.001	.315–1.377	0.43
		dfn=3	ChatGPT vs. Co-Pilot	.501	.201	.087	–.041–1.044	0.22
		dfd=1,095	Gemini vs. DeepSeek	2.415	.185	<.001	1.916–2.914	1.58
		MS = 123.427	Gemini vs. Co-Pilot	2.070	.193	<.001	1.549–2.591	1.25
		*P* < .001	DeepSeek vs. Co-Pilot	–.345	.132	.063	–.701–.011	0.29
		*η*² = 0.390						
	^a^Question category (main effects)	*F* = 0.495	1 vs. 2	.363	.4,131	1.000	–.750–1.476	0.19
		df = 3	1 vs. 3	–.032	.3995	1.000	–1.109–1.044	0.02
		dfn=3	1 vs. 4	.210	.3794	1.000	–.812–1.233	0.10
		dfd=365	2 vs. 3	–.395	.3642	1.000	–1.376–.586	0.22
		MS = 3.129	2 vs. 4	–.152	.3421	1.000	–1.074–.769	0.07
		*P* = .687	3 vs. 4	.243	.3255	1.000	–.635–1.120	0.12
		*η*^2^ = 0.015						
	AI chatbot-Question category (interaction effects)	*F* = 1.410						
		df = 7.592^b^						
		dfn=9						
		dfd=1,095						
		MS = 2.867						
		*P* = .197						
		*η*^2^ = 0.043						
SMOG scores	AI Chatbot (main effects)	*F* = 68.979	ChatGPT vs. Gemini	–1.488	.190	<.001	–1.999––.977	0.96
		df = 2.500^b^	ChatGP vs. DeepSeek	.307	.161	.353	–.126–.740	018
		dfn = 3	ChatGPT vs. Co-Pilot	.580	.177	.009	.103–1.057	0.37
		dfd = 1,095	Gemini vs. DeepSeek	1.795	.142	<.001	1.412–2.179	1.51
		MS = 93.425	Gemini vs. Co-Pilot	2.068	.152	<.001	1.659–2.477	1.66
		*P* < .001	DeepSeek vs. Co-Pilot	.272	.108	.082	–.020–.564	0.30
		*η*^2^ = 0.421						
	^a^Question category (main effects)	*F* = 0.257	1 vs. 2	.230	.312	1.000	–.610–1.069	016
		df = 3	1 vs. 3	.018	.301	1.000	–.794–.830	0.01
		dfn = 3	1 vs. 4	.091	.286	1.000	–.680–.863	0.05
		dfd = 365	2 vs. 3	–.212	.275	1.000	–.952–.529	0.15
		MS = 0.924	2 vs. 4	–.138	.258	1.000	–.833–.557	0.09
		*P* = .856	3 vs. 4	.073	.246	1.000	–.588–.735	0.04
		*η*^2^ = 0.008						
	AI chatbot-Question category (interaction effects)	*F* = 1.368						
		df = 7.501^b^						
		dfn = 9						
		dfd=1,095						
		MS = 1.853						
		*P* = .215						
		*η*^2^ = 0.041						

a Question categories: 1= Biological and surgical aspects, 2= Prosthetic and material science, 3= Clinical outcomes and complications, and 4= Patient centered and economic factors.

b Greenhouse-Geisser adjustment for degrees of freedom was used as Mauchly test of sphericity was significant (*P* < .001).

F, F statistic; df, Degree of freedom; dfn, Numerator degree of freedom; dfd, Denominator degree of freedom; MS, Mean square; P, Probability value; *η*^2^, Partial Eta squared; vs., Versus; MD, Mean difference; SE, Standard error; CI, 95% confidence intervals of mean difference; ES, Cohen's d Effect size.

Nevertheless, no significant differences in FKG scores were found between different question categories (main effects: F(3) = 0.495, *P* = .687, effect size *η*^2^ = 0.015, Table 3). Moreover, no significant interaction effects of different AI chatbots and question categories on the variations in FKG scores were detected (interaction effects: F(7.592) = 1.410, *P* = .197, effect size *η*^2^ = 0.043, Table 3). Figure 3 demonstrates the profile plots of FKG scores based on AI chatbot and category of questions.

**Figure 3:**
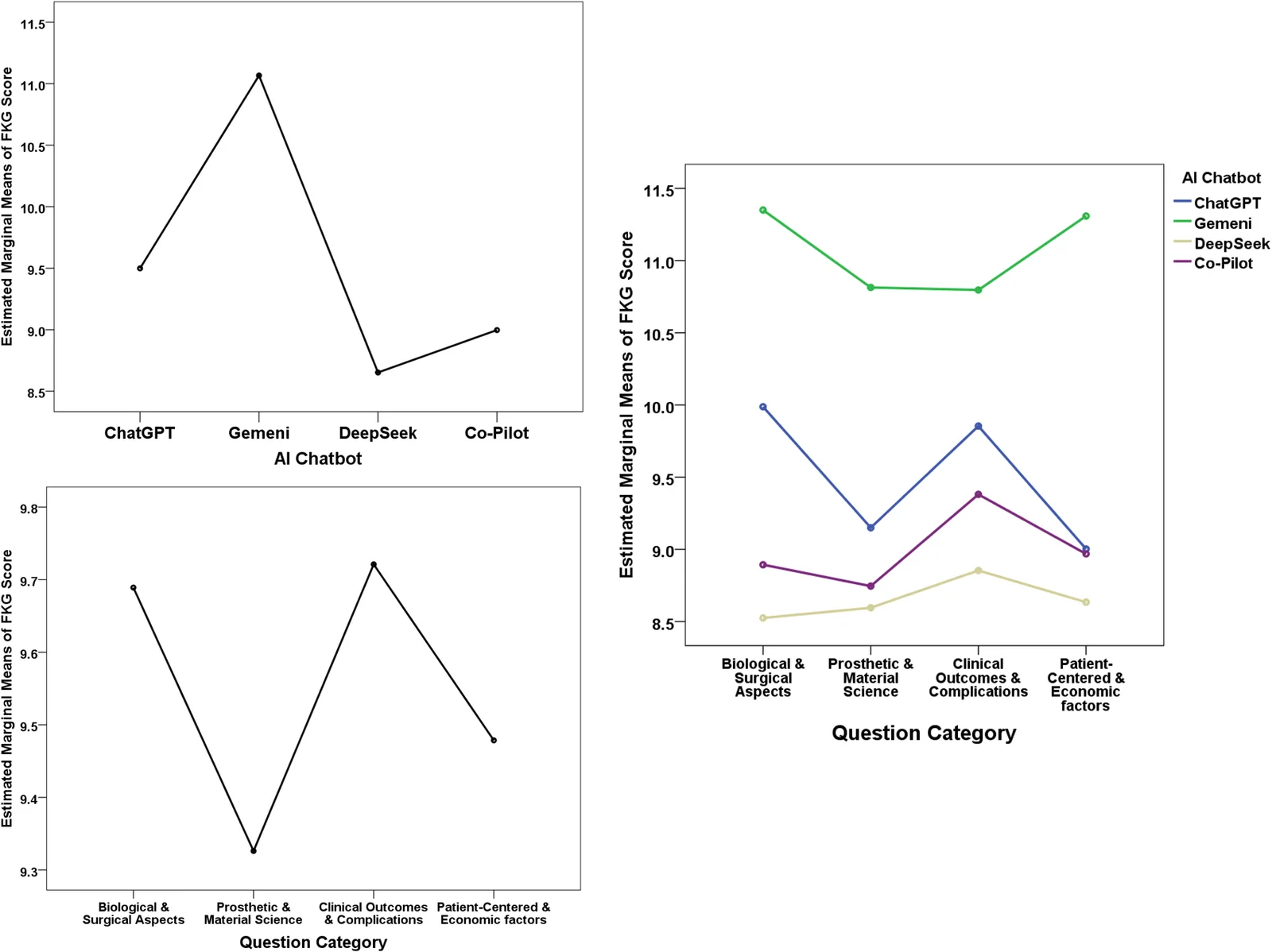
The profile plots of FKG scores based on AI chatbot and category of questions (*n* = 99).

Similarly, significant differences were found in SMOG scores between the tested AI chatbots in this study (main effects: F(2.500) = 68.979, *P* < .001, effect size *η*^2^ = 0.421). This indicated that the choice of AI chatbot strongly and reliably explains a large portion of the differences in SMOG scores (42% of the differences). Further pairwise comparisons showed that Gemini AI chatbot was associated with higher SMOG scores (i.e., more difficult to read answers) than ChatGPT, DeepSeek, and Co-Pilot AI chatbots (*P* < .001, Table 3). Also, ChatGPT AI chatbot had higher SMOG scores (i.e., more difficult to read answers) than Co-Pilot AI chatbot (*P* = .009, Table 3).

Nevertheless, no significant differences in SMOG scores were found between different question categories (main effects: F(3) = 0.257, *P* = .856, effect size *η*^2^ = 0.008, Table 3). Moreover, no significant interaction effects of different AI chatbots and question categories on the variations in SMOG scores were detected (interaction effects: F(7.501) = 1.368, *P* = .215, effect size *η*^2^ = 0.041, Table 3). Figure 4 demonstrates the profile plots of SMOG scores based on AI chatbot and category of questions.

**Figure 4:**
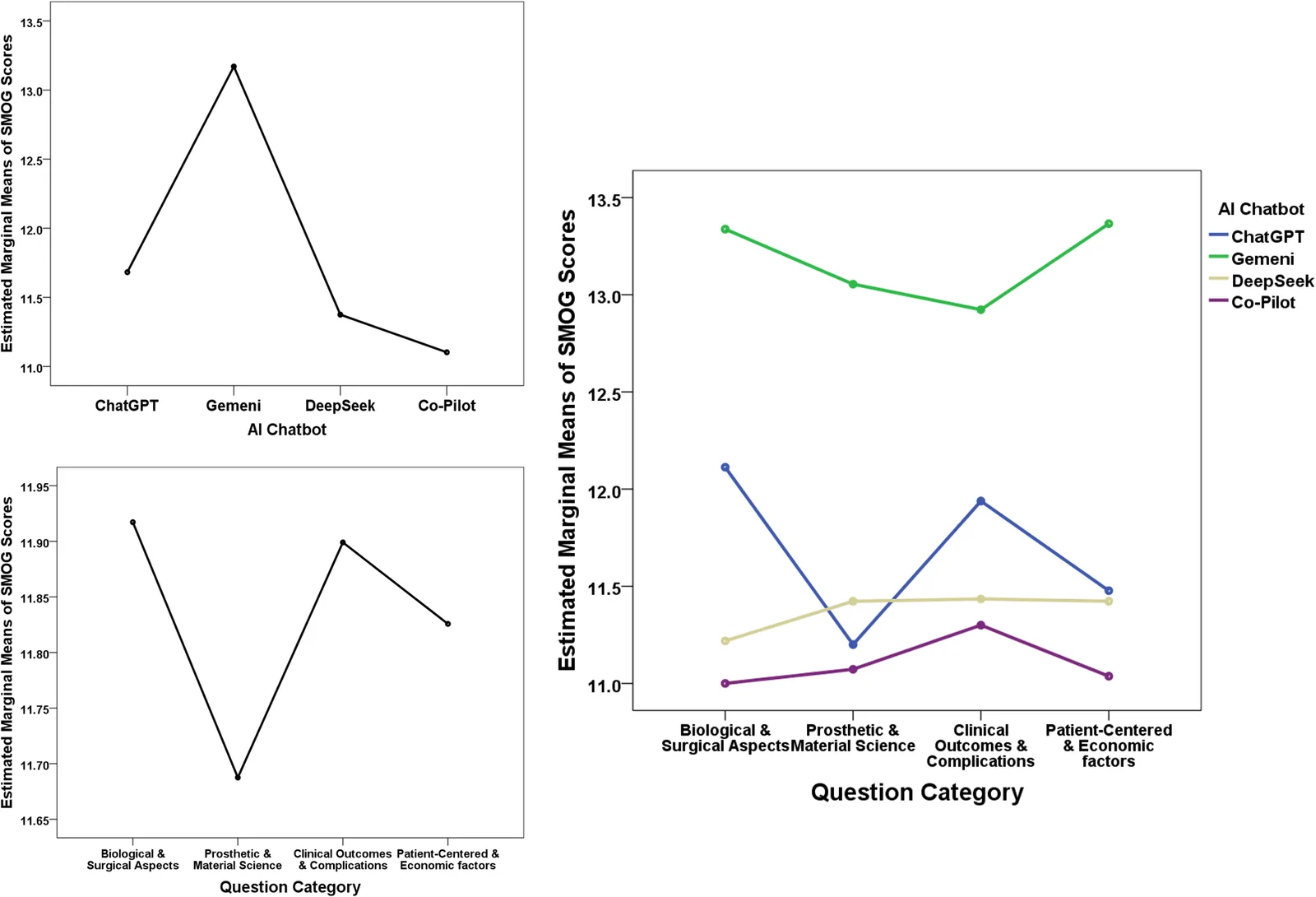
The profile plots of SMOG scores based on AI chatbot and category of questions (*n* = 99).

### Usefulness of AI chatbot generated responses

Considering all questions as well as each question category, the majority of the AI chatbot generated responses were either “useful” or “partially useful”, except for the DeepSeek AI chatbot which has most responses either “partially useful” or “not useful” (Table 2 and Figure 5). Considering all questions together, the usefulness scores were significantly different between AI chatbots (*P* < .001, effect size *w* = 0.310, Table 4). This indicated that the type of AI chatbot strongly and reliably explains the differences in usefulness scores, with a medium to large effect size (explains more than 30% of the differences).

**Figure 5:**
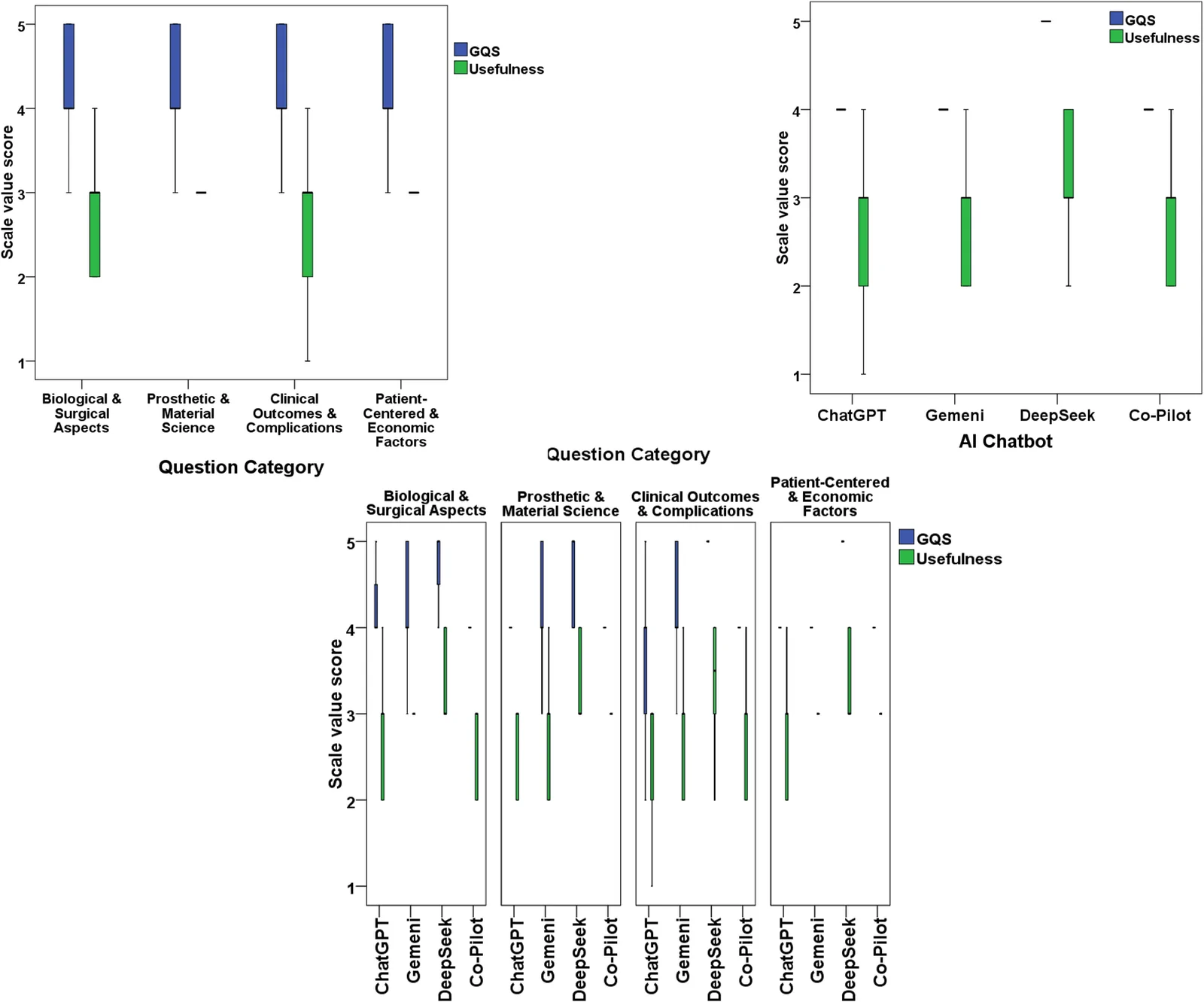
Boxplots of the distribution of usefulness and GQS scores based on AI chatbot and category of questions (*n* = 99).

**Table 4:** Differences in usefulness, GQS, and total CLEAR scores based on different AI chatbots and question categories (*n* = 99 questions).

Dependant variable	^a^Factor	Friedman test	Wilcoxon signed ranks test between pairs				
			Compared pairs	*Z*	*P*	*Q*	*r*
Usefulness score	AI chatbot in all questions	X^2^ = 92.080	ChatGPT vs. Gemini	−2.413	.016	0.033	0.029
		df = 3	ChatGPT vs. DeepSeek	–6.813	<.001	0.008	0.234
		*P* < .001	ChatGPT vs. Co-Pilot	–2.091	.037	0.042	0.022
		*W* = 0.310	Gemini vs. DeepSeek	–6.286	<.001	0.017	0.200
			Gemini vs. Co-Pilot	–.149	.881	0.050	0.000
			DeepSeek vs. Co-Pilot	–6.689	<.001	0.025	0.226
	AI chatbot in question category 1	X^2^ = 19.418	ChatGPT vs. Gemini	–1.000	.317	0.042	0.031
		df = 3	ChatGPT vs. DeepSeek	–2.495	.013	0.025	0.195
		*P* < .001	ChatGPT vs. Co-Pilot	–.632	.527	0.050	0.012
		W = 0.405	Gemini vs. DeepSeek	–2.530	.011	0.017	0.200
			Gemini vs. Co-Pilot	–2.236	.025	0.033	0.156
			DeepSeek vs. Co-Pilot	–3.357	.001	0.008	0.352
	AI chatbot in question category 2	X^2^ = 18.900	ChatGPT vs. Gemini	–1.508	.132	0.042	0.052
		df = 3	ChatGPT vs. DeepSeek	–3.176	.001	0.008	0.229
		*P* < .001	ChatGPT vs. Co-Pilot	–2.309	.021	0.033	0.121
		*W* = 0.286	Gemini vs. DeepSeek	–2.653	.008	0.017	0.160
			Gemini vs. Co-Pilot	–1.000	.317	0.050	0.023
			DeepSeek vs. Co-Pilot	–2.530	.011	0.025	0.145
	AI chatbot in question category 3	X^2^ = 30.610	ChatGPT vs. Gemini	–1.069	.285	0.033	0.022
		df = 3	ChatGPT vs. DeepSeek	–3.750	<.001	0.008	0.270
		*P* < .001	ChatGPT vs. Co-Pilot	–.871	.384	0.042	0.015
		*W* = 0.392	Gemini vs. DeepSeek	–4.066	<.001	0.017	0.318
			Gemini vs. Co-Pilot	.000	1.000	0.050	0.000
			DeepSeek vs. Co-Pilot	–3.704	<.001	0.025	0.264
	AI chatbot in question category 4	X^2^ = 29.733	ChatGPT vs. Gemini	–1.321	.186	0.042	0.025
		df = 3	ChatGPT vs. DeepSeek	–4.099	<.001	0.008	0.240
		*P* < .001	ChatGPT vs. Co-Pilot	–1.528	.127	0.033	0.033
		*W* = 0.283	Gemini vs. DeepSeek	–3.346	.001	0.025	0.160
			Gemini vs. Co-Pilot	–.258	.796	0.050	0.001
			DeepSeek vs. Co-Pilot	–3.755	<.001	0.017	0.201
Global Quality Scale score	AI chatbot in all questions	X^2^ = 142.637	ChatGPT vs. Gemini	–2.874	.004	0.033	0.042
		df = 3	ChatGPT vs. DeepSeek	–7.614	<.001	0.008	0.293
		*P* < .001	ChatGPT vs. Co-Pilot	–.752	.452	0.050	0.003
		*W* = 0.480	Gemini vs. DeepSeek	–7.221	<.001	0.017	0.263
			Gemini vs. Co-Pilot	–2.536	.011	0.042	0.032
			DeepSeek vs. Co-Pilot	–8.328	<.001	0.025	0.350
	AI chatbot in question category 1	X^2^ = 17.06	ChatGPT vs. Gemini	–.447	.655	0.050	0.006
		df = 3	ChatGPT vs. DeepSeek	–2.496	.013	0.025	0.195
		*P* = .001	ChatGPT vs. Co-Pilot	–1.000	.317	0.042	0.031
		*W* = 0.355	Gemini vs. DeepSeek	–2.530	.011	0.017	0.200
			Gemini vs. Co-Pilot	–1.134	.257	0.033	0.040
			DeepSeek vs. Co-Pilot	–3.317	.001	0.008	0.344
	AI chatbot in question category 2	X^2^ = 20.366	ChatGPT vs. Gemini	–1.508	.132	0.033	0.052
		df = 3	ChatGPT vs. DeepSeek	–3.259	.001	0.008	0.241
		*P* < .001	ChatGPT vs. Co-Pilot	–.586	.558	0.050	0.008
		*W* = 0.309	Gemini vs. DeepSeek	–2.673	.008	0.025	0.162
			Gemini vs. Co-Pilot	–.905	.366	0.042	0.019
			DeepSeek vs. Co-Pilot	–3.153	.002	0.017	0.226
	AI chatbot in question category 3	X^2^ = 54.399	ChatGPT vs. Gemini	–3.035	.002	0.033	0.177
		df = 3	ChatGPT vs. DeepSeek	–4.434	<.001	0.008	0.378
		*P* < .001	ChatGPT vs. Co-Pilot	–1.500	.134	0.050	0.043
		*W* = 0.697	Gemini vs. DeepSeek	–4.146	<.001	0.017	0.331
			Gemini vs. Co-Pilot	–2.310	.021	0.042	0.103
			DeepSeek vs. Co-Pilot	–4.772	<.001	0.025	0.438
	AI chatbot in question category 4	X^2^ = 65.458	ChatGPT vs. Gemini	.000	1.000	0.050	0.000
		df = 3	ChatGPT vs. DeepSeek	–4.691	<.001	0.008	0.314
		*P* < .001	ChatGPT vs. Co-Pilot	–.302	.763	0.042	0.001
		*W* = 0.623	Gemini vs. DeepSeek	–4.691	<.001	0.017	0.314
			Gemini vs. Co-Pilot	–.378	.705	0.033	0.002
			DeepSeek vs. Co-Pilot	–5.112	<.001	0.025	0.373
Total CLEAR Reliability score	AI chatbot in all questions	X^2^ = 115.970	ChatGPT vs. Gemini	–.699	.484	0.050	0.002
		df = 3	ChatGPT vs. DeepSeek	–7.305	<.001	0.008	0.270
		*P* < .001	ChatGPT vs. Co-Pilot	–2.383	.017	0.033	0.029
		*W* = 0.390	Gemini vs. DeepSeek	–6.734	<.001	0.017	0.229
			Gemini vs. Co-Pilot	–1.479	.139	0.042	0.011
			DeepSeek vs. Co-Pilot	–7.264	<.001	0.025	0.266
	AI chatbot in question category 1	X^2^ = 18.568	ChatGPT vs. Gemini	–1.274	.203	0.033	0.051
		df = 3	ChatGPT vs. DeepSeek	–3.001	.003	0.017	0.281
		*P* < .001	ChatGPT vs. Co-Pilot	–1.042	.298	0.042	0.034
		*W* = 0.387	Gemini vs. DeepSeek	–2.716	.007	0.025	0.231
			Gemini vs. Co-Pilot	–.282	.778	0.050	0.002
			DeepSeek vs. Co-Pilot	–3.272	.001	0.008	0.335
	AI chatbot in question category 2	X^2^ = 21.814	ChatGPT vs. Gemini	–.712	.476	0.050	0.012
		df = 3	ChatGPT vs. DeepSeek	–2.955	.003	0.017	0.198
		*P* < .001	ChatGPT vs. Co-Pilot	–.835	.403	0.033	0.016
		*W* = 0.331	Gemini vs. DeepSeek	–2.895	.004	0.025	0.190
			Gemini vs. Co-Pilot	–.796	.426	0.042	0.014
			DeepSeek vs. Co-Pilot	–3.084	.002	0.008	0.216
	AI chatbot in question category 3	X^2^ = 42.200	ChatGPT vs. Gemini	–1.377	.169	0.042	0.036
		df = 3	ChatGPT vs. DeepSeek	–4.268	<.001	0.008	0.350
		*P* < .001	ChatGPT vs. Co-Pilot	–1.414	.157	0.033	0.038
		*W* = 0.541	Gemini vs. DeepSeek	–3.668	<.001	0.017	0.259
			Gemini vs. Co-Pilot	–.159	.873	0.050	0.000
			DeepSeek vs. Co-Pilot	–4.226	<.001	0.025	0.343
	AI chatbot in question category 4	X^2^ = 38.206	ChatGPT vs. Gemini	–.870	.384	0.050	0.011
		df = 3	ChatGPT vs. DeepSeek	–4.415	<.001	0.008	0.278
		*P* < .001	ChatGPT vs. Co-Pilot	–1.422	.155	0.042	0.029
		*W* = 0.364	Gemini vs. DeepSeek	–4.237	<.001	0.017	0.256
			Gemini vs. Co-Pilot	–2.032	**.** **042** ^b^	0.033	0.059
			DeepSeek vs. Co-Pilot	–3.951	<.001	0.025	0.223

a Question categories: 1, Biological and surgical aspects; 2, Prosthetic and material science; 3, Clinical outcomes and complications; 4, Patient centered and economic factors; CLEAR, Completeness; Lack of false information; Evidence support; Appropriateness; and Relevance reliability tool; X^2^, Chi square statistic utilizing the Friedman test; df, Degree of freedom; W, Effect size Kendall's W for Friedman test; Z, Z statistic; P, Probability value; Q, Benjamini-Hochberg critical value for adjustment to multiple comparisons based on False Discovery Rate (FDR) of 5%; and a *P* value below the Q value is considered significant; r, Effect size r for Wilcoxon signed ranks test.

b P value is considered not significant after Benjamini-Hochberg adjustment for multiple comparisons based on False Discovery Rate (FDR) of 5%.

ChatGPT was associated with more useful AI generated responses (indicated by significantly lower usefulness scores) than other AI chatbots (*P* < .05, Table 4). Also, DeepSeek was associated with less useful AI generated responses (indicated by higher usefulness scores) than Gemini (*P* < .001, Effect size *r* = 0.200) and Co-Pilot (*P* < .001, *r* = 0.226) (Table 4). This indicated that type of AI chatbot strongly and reliably explains the differences in usefulness scores, with a medium effect size (more than 20% of the differences).

Considering the effect of category of question regardless the type of AI chatbot, no significant differences were found in usefulness scores between different question categories (Kruskal Wallis X^2^(3) = 1.320, *P* = .724).

### Quality of AI chatbot generated responses

Considering all questions together as well as each question category, the median GQS score was 4 except for DeepSeek AI chatbot it was 5 (Supplementary Table S1). The majority of AI generated responses were of “good” or “excellent” quality considering all questions together as well as within each question category (Table 2 and Figure 5). The GQS scores were significantly different between AI chatbots considering all questions together (*P* < .001, effect size *w* = 0.480, Table 4) as well as within each question category (*P* < .001, Table 4). This indicated that the type of AI chatbot strongly and reliably explains the differences in GQS scores, with a large effect size (explains 48% of the differences). DeepSeek was associated with more “excellent” quality AI generated responses than other tested AI chatbots for all questions together as well as within each question category (*P* < .05, Table 4). Considering the effect of the category of question regardless the type of AI chatbot, no significant differences were found in GQS scores between different question categories (Kruskal Wallis X^2^(3) = 1.842, *P* = .606).

### Reliability of AI chatbot generated responses

The CLEAR reliability scores ranged between 19 and 25 with a median between 22.5 and 25 based on the AI chatbot and question category (Table 1). All AI generated responses were of “very good” content considering all questions together as well as within each question category (Table 2 and Figure 5). The CLEAR scores were significantly different between AI chatbots considering all questions together (*P* < .001, effect size *w* = 0.390, Table 4) as well as within each question category (*P* < .001, Table 4). This indicated that the type of AI chatbot strongly and reliably explains the differences in CLEAR scores, with a large effect size (explains 39% of the differences). DeepSeek was associated with higher total CLEAR scores for the AI generated responses (median = 25) than other tested AI chatbots for all questions together as well as within each question category (*P* < .05, Table 4). Considering the effect the category of question regardless the type of AI chatbot, significant differences were found in CLEAR scores between different question categories (Kruskal Wallis X^2^(3) = 9.318, *P* = .025, effect size *η*^2^ = 0.016). This indicated that the category of question would reliably explain the differences in CLEAR scores, with only a small effect size (explains only 1.6% of the differences) and minimal practical impacts. Further pairwise comparisons showed that patient centered and economic question category was associated with higher CLEAR scores (i.e., more reliable content) than prosthetic and material science (MWU = 4,829.5, *Z* = −2.887, *P* = .004, effect size *r* = 0.146) and clinical outcomes and complications (MWU=6,242.0, *Z* = −2.021, *P* = .043, *r* = 0.067) question categories. This indicates a significant tendency for patient-centered and economic topics to provide more reliable information than other topics. However, the overall magnitude of the difference in the population is relatively modest and has minimal practical impacts (only explains 6.7 to 14.6% of the difference).

### Correlations between variables

Higher FKG scores (i.e., more difficult to read answers) were strongly associated with higher SMOG scores (i.e., more difficult to read answers) within each AI chatbot (*P* < .001, Pearson *r* ≥ .898, Supplementary Table S3).

Higher FKG as well as SMOG scores of the generated responses (i.e., more difficult to read answers) were associated with lower GQS scores (worse quality) in Gemini AI chatbot, lower usefulness scores (more useful response) in ChatGPT, and lower CLEAR scores (less reliable content) in Gemini and Co-Pilot (*P* < .050, Supplementary Table S4).

Besides, positive correlations were found between GQS, usefulness, and CLEAR scores within each AI chatbot. Because the usefulness scale is inverted, this positive correlation indicates that as quality and reliability scores increased, the usefulness scores also numerically increased—meaning the responses were rated as less useful (*P* ≤ .001, Supplementary Table S4).

## Discussion

This study evaluated the performance of several artificial intelligence (AI) chatbots in answering public questions related to dental implants, focusing on reliability, quality, readability, and usefulness. Overall, all models generated responses that were rated favorably in terms of reliability, quality, readability, and usefulness, highlighting the potential of AI chatbots as supportive sources of information in implant dentistry.

A major concern with using artificial intelligence (AI) for patient education is the risk of inaccurate or fabricated information, known as AI hallucinations. These errors can lead to unrealistic patient expectations, unnecessary anxiety, and reduced trust between patients and clinicians (24, 28). To assess the reliability of AI-generated responses, this study used the CLEAR framework for clinical information, which evaluates Completeness, lack of false information, evidence support, appropriateness, and relevance (29). Overall, all AI models performed very well, with median CLEAR scores falling within the highest category of very good content (19–24, 29). This suggests that current chatbots provide reliable information on implant dentistry that is complete, relevant, and mostly free from false information. Similar findings have been reported in previous dental studies evaluating AI-generated responses in endodontics, orthodontics, dental trauma, crown restorations, and oral cancer education (7, 17–20).

Among the models evaluated, DeepSeek achieved the highest overall performance, with a mean CLEAR score of 24.68 and a median score of 25. It consistently produced accurate responses across biological, surgical, prosthetic, and complication-related questions and significantly outperformed ChatGPT, Gemini, and Copilot. These findings agree with recent studies showing that DeepSeek provides more guideline-consistent responses and stronger clinical reasoning than several other AI models, including ChatGPT-4o, Claude, and Copilot (22, 30).

In the lack of false information domain, DeepSeek and Copilot both achieved a median score of 5, indicating minimal hallucinations or fabricated clinical information. This is encouraging given previous concerns regarding AI-generated medical misinformation (24). Similar findings have been reported in studies on cosmetic tooth whitening, dental trauma, and pediatric endodontics, where modern AI chatbots generally avoided providing false information and frequently advised users to seek professional dental care (23, 30). Overall, these results suggest that the evaluated AI models demonstrated a low tendency to generate false clinical information within the scope of the present question set. Despite acceptable performance scores, a key limitation of generative large language models is the lack of consistent evidence traceability. Responses are generated without direct linkage to verifiable source material, which may limit independent validation of clinical statements. This limitation has been widely reported in evaluations of AI systems in healthcare, where hallucinations and non-citable outputs remain a concern (24, 25). Although the CLEAR tool provides a structured method for evaluating AI-generated health information, its scores should not be interpreted as evidence of clinical validation or safety for individual patient care. The concentration of scores near the upper limit with a very good category for all AI systems tested leads to the loss of tool power in differentiating between AI systems. Another concern about the CLEAR tool relies in the fact that it was validated by a small number of healthcare professionals with no other tool to compare it with (25), which might questionings the validity of using this tool alone in comparison.

Therefore, evaluation should rely on multiple assessments tools. Although all chatbots showed high reliability, differences were observed in response quality. DeepSeek consistently achieved higher GQS scores and a greater proportion of “excellent” responses compared with the other models. This may reflect differences in response structure and depth across models. Previous studies have also reported variability in AI model performance depending on topic complexity and evaluation criteria (10, 18, 19, 22, 30). In contrast, usefulness analysis showed that ChatGPT achieved better usability (indicated by lower usefulness scores) despite lower CLEAR scores, whereas DeepSeek, although more comprehensive, was perceived as a less user-friendly form (reflected by its higher usefulness scores). This suggests that higher informational depth does not necessarily translate into improved patient-centered communication (7, 8, 10). The observed pattern highlights a potential trade-off between comprehensiveness and usability, where more technical and detailed responses may reduce patient comprehension.

Readability analysis further supported these findings. The interpretation of readability scores is important in dental care, Patient education materials should be presented in a clear and easy way to accommodate differences in health literacy. The American Medical Association (AMA) and the National Institutes of Health (NIH) recommend that such materials be written at approximately a 6th- to 8th-grade reading level to ensure they are accessible to the general population (31).

All models exceeded the recommended 6th–8th grade reading level for patient education, as suggested in health literacy literature. Gemini produced the most difficult outputs based on FKG and SMOG indices, while DeepSeek and Copilot generated comparatively more readable content. However, even the lowest-scoring outputs remained above optimal thresholds for general patient education.

A primary limitation of readability scores, is that they assess mainly the structural and syntactic complexity of a text, such as syllable count and sentence length, rather than directly measuring actual patient comprehension. True comprehension is a multifactorial process that can be influenced by individual patient characteristics, including health literacy, educational background, prior knowledge, and familiarity with dental terminology. Nevertheless, readability remains an important consideration in patient education. Poor readability may make implant-related information more difficult to understand and could potentially influence patients' understanding of the proposed treatment, expectations, and treatment-related decisions. Implant dentistry involves inherently complex terminology (e.g., osseointegration, peri-implantitis, sinus augmentation), which increases cognitive and linguistic burden. Consistent with previous literature on AI-generated patient education and implant-related chatbot evaluations, variability in readability across models has been reported (32, 33). Effective dentist–patient communication is essential for treatment understanding and informed decision-making (34). This highlights the importance of balancing scientific accuracy with accessibility in AI-generated clinical information. These findings should also be interpreted in the context of differences in question type and evaluation design across studies. Altuğ Yıldırım and Alim (36), who evaluated DUS questions, reported a significant difference between models in paediatric dentistry but not in restorative dentistry. Such differences highlight the challenges of directly comparing studies using different model versions and question formats. Answering a multiple-choice examination question correctly differs from responding to an open-ended patient query, where accuracy, comprehensibility, relevance, and overall quality of health information are also important considerations.

Model performance remained relatively stable across question categories, although patient-centered questions tended to yield slightly higher reliability scores than more technically demanding prosthetic and complication-related topics.

From an educational perspective, AI chatbots may serve as supplementary sources of information for patients seeking information about dental implants. However, AI-generated content should not replace professional judgment. Current systems may provide incomplete or generalized recommendations that do not account for individual clinical variables such as bone quality, systemic health, or radiographic findings. Implant treatment planning requires comprehensive clinical assessment and cannot be fully automated at present. Therefore, AI should be considered an adjunct educational tool within a clinician-supervised, human-in-the-loop framework.

This study has several limitations. AI model outputs may vary over time due to continuous updates. Only English-language responses were evaluated, which may limit generalizability. Although standardized scoring systems were used, some subjectivity in expert evaluation is unavoidable. Additionally, patient-based assessments were not included, and the variability in repeated queries was not assessed. Future research should evaluate newer models, multilingual performance, variability in repeated queries, and patient-centered outcomes. Development of domain-specific dental AI systems trained on validated implant dentistry literature, may further improve reliability, transparency, and clinical applicability. Future studies could investigate whether differences in AI-generated responses translate into differences in patient understanding, confidence, or anxiety, as these outcomes were not assessed in the present study (35, 36).

Conclusions: AI-powered chatbots can provide generally reliable and high-quality information regarding dental implants, demonstrating their potential as supplementary patient education tools. However, significant differences exist among AI models; notably, models generating the most highly reliable content often produce less readable and less user-friendly responses. Given these readability barriers and the lack of direct clinical validation, AI-generated information must complement, rather than replace, professional dental consultation. Further studies involving direct patient assessments and clinically validated outcomes are warranted.

## Data Availability

The datasets presented in this study can be found in online repositories. The names of the repository/repositories and accession number(s) can be found in the article/Supplementary Material.
