# Supplementary material for: The use of different artificial intelligence-powered chatbots to answer questions related to dental implants
Source: Front Oral Health. 2026 Oct 21;7:1975960. doi: 10.3389/froh.2026.1975960 (PMC13731212; doi:10.3389/froh.2026.1975960)
Supplement: Supplementary file 1 [file Table1.docx]

**Supplementary Table 1.** Distribution of CLEAR individual element, GQS, and usefulness scores based on AI chatbot and category of questions in this study (n= 99 questions).

| Variable | Statistic | AI Chatbot | ^$^Question Category | | | | |
| --- | --- | --- | --- | --- | --- | --- | --- |
|  |  |  | All (n=99) | 1 (n=16) | 2 (n=22) | 3 (n=26) | 4 (n=35) |
| CLEAR Completeness score | Mean | ChatGPT | 3.91 | 3.81 | 3.82 | 3.81 | 4.09 |
|  | SD |  | .573 | .834 | .588 | .491 | .445 |
|  | Median |  | 4.00 | 4.00 | 4.00 | 4.00 | 4.00 |
|  | Min |  | 2 | 3 | 2 | 2 | 3 |
|  | Max |  | 5 | 5 | 5 | 4 | 5 |
|  | IQR |  | 0 | 2 | 0 | 0 | 0 |
|  | Mean | Gemini | 4.21 | 4.38 | 4.05 | 4.27 | 4.20 |
|  | SD |  | .520 | .619 | .575 | .452 | .473 |
|  | Median |  | 4.00 | 4.00 | 4.00 | 4.00 | 4.00 |
|  | Min |  | 3 | 3 | 3 | 4 | 3 |
|  | Max |  | 5 | 5 | 5 | 5 | 5 |
|  | IQR |  | 1 | 1 | 0 | 1 | 0 |
|  | Mean | DeepSeek | 4.81 | 4.75 | 4.68 | 4.96 | 4.80 |
|  | SD |  | .421 | .447 | .568 | .196 | .406 |
|  | Median |  | 5.00 | 5.00 | 5.00 | 5.00 | 5.00 |
|  | Min |  | 3 | 4 | 3 | 4 | 4 |
|  | Max |  | 5 | 5 | 5 | 5 | 5 |
|  | IQR |  | 0 | 1 | 1 | 0 | 0 |
|  | Mean | Co-Pilot | 4.05 | 3.81 | 4.00 | 3.96 | 4.26 |
|  | SD |  | .542 | .544 | .617 | .445 | .505 |
|  | Median |  | 4.00 | 4.00 | 4.00 | 4.00 | 4.00 |
|  | Min |  | 3 | 3 | 3 | 3 | 3 |
|  | Max |  | 5 | 5 | 5 | 5 | 5 |
|  | IQR |  | 0 | 1 | 0 | 0 | 1 |
| CLEAR Lack of False score | Mean | ChatGPT | 4.66 | 4.75 | 4.59 | 4.50 | 4.77 |
|  | SD |  | .538 | .577 | .590 | .583 | .426 |
|  | Median |  | 5.00 | 5.00 | 5.00 | 5.00 | 5.00 |
|  | Min |  | 3 | 3 | 3 | 3 | 4 |
|  | Max |  | 5 | 5 | 5 | 5 | 5 |
|  | IQR |  | 1 | 0 | 1 | 1 | 0 |
|  | Mean | Gemini | 4.80 | 4.88 | 4.73 | 4.73 | 4.86 |
|  | SD |  | .404 | .342 | .456 | .452 | .355 |
|  | Median |  | 5.00 | 5.00 | 5.00 | 5.00 | 5.00 |
|  | Min |  | 4 | 4 | 4 | 4 | 4 |
|  | Max |  | 5 | 5 | 5 | 5 | 5 |
|  | IQR |  | 0 | 0 | 1 | 1 | 0 |
|  | Mean | DeepSeek | 4.92 | 4.94 | 4.82 | 4.88 | 5.00 |
|  | SD |  | .274 | .250 | .395 | .326 | .000 |
|  | Median |  | 5.00 | 5.00 | 5.00 | 5.00 | 5.00 |
|  | Min |  | 4 | 4 | 4 | 4 | 5 |
|  | Max |  | 5 | 5 | 5 | 5 | 5 |
|  | IQR |  | 0 | 0 | 0 | 0 | # |
|  | Mean | Co-Pilot | 4.70 | 4.75 | 4.68 | 4.62 | 4.74 |
|  | SD |  | .462 | .447 | .477 | .496 | .443 |
|  | Median |  | 5.00 | 5.00 | 5.00 | 5.00 | 5.00 |
|  | Min |  | 4 | 4 | 4 | 4 | 4 |
|  | Max |  | 5 | 5 | 5 | 5 | 5 |
|  | IQR |  | 1 | 1 | 1 | 1 | 1 |
| CLEAR Evidence score | Mean | ChatGPT | 4.94 | 4.94 | 4.91 | 4.92 | 4.97 |
|  | SD |  | .240 | .250 | .294 | .272 | .169 |
|  | Median |  | 5.00 | 5.00 | 5.00 | 5.00 | 5.00 |
|  | Min |  | 4 | 4 | 4 | 4 | 4 |
|  | Max |  | 5 | 5 | 5 | 5 | 5 |
|  | IQR |  | 0 | 0 | 0 | 0 | 0 |
|  | Mean | Gemini | 4.92 | 4.88 | 4.91 | 4.92 | 4.94 |
|  | SD |  | .274 | .342 | .294 | .272 | .236 |
|  | Median |  | 5.00 | 5.00 | 5.00 | 5.00 | 5.00 |
|  | Min |  | 4 | 4 | 4 | 4 | 4 |
|  | Max |  | 5 | 5 | 5 | 5 | 5 |
|  | IQR |  | 0 | 0 | 0 | 0 | 0 |
|  | Mean | DeepSeek | 4.98 | 5.00 | 4.95 | 4.96 | 5.00 |
|  | SD |  | .141 | .000 | .213 | .196 | .000 |
|  | Median |  | 5.00 | 5.00 | 5.00 | 5.00 | 5.00 |
|  | Min |  | 4 | 5 | 4 | 4 | 5 |
|  | Max |  | 5 | 5 | 5 | 5 | 5 |
|  | IQR |  | 0 | # | 0 | 0 | # |
|  | Mean | Co-Pilot | 4.95 | 4.94 | 4.86 | 4.96 | 5.00 |
|  | SD |  | .220 | .250 | .351 | .196 | .000 |
|  | Median |  | 5.00 | 5.00 | 5.00 | 5.00 | 5.00 |
|  | Min |  | 4 | 4 | 4 | 4 | 5 |
|  | Max |  | 5 | 5 | 5 | 5 | 5 |
|  | IQR |  | 0 | 0 | 0 | 0 | # |
| CLEAR Appropriateness score | Mean | ChatGPT | 4.84 | 4.56 | 4.77 | 4.96 | 4.91 |
|  | SD |  | .422 | .512 | .528 | .196 | .373 |
|  | Median |  | 5.00 | 5.00 | 5.00 | 5.00 | 5.00 |
|  | Min |  | 3 | 4 | 3 | 4 | 3 |
|  | Max |  | 5 | 5 | 5 | 5 | 5 |
|  | IQR |  | 0 | 1 | 0 | 0 | 0 |
|  | Mean | Gemini | 4.56 | 4.56 | 4.36 | 4.65 | 4.60 |
|  | SD |  | .772 | .814 | .953 | .745 | .651 |
|  | Median |  | 5.00 | 5.00 | 5.00 | 5.00 | 5.00 |
|  | Min |  | 3 | 3 | 3 | 3 | 3 |
|  | Max |  | 5 | 5 | 5 | 5 | 5 |
|  | IQR |  | 1 | 1 | 2 | 0 | 1 |
|  | Mean | DeepSeek | 4.98 | 5.00 | 4.95 | 5.00 | 4.97 |
|  | SD |  | .141 | .000 | .213 | .000 | .169 |
|  | Median |  | 5.00 | 5.00 | 5.00 | 5.00 | 5.00 |
|  | Min |  | 4 | 5 | 4 | 5 | 4 |
|  | Max |  | 5 | 5 | 5 | 5 | 5 |
|  | IQR |  | 0 | # | 0 | # | 0 |
|  | Mean | Co-Pilot | 4.98 | 5.00 | 4.91 | 5.00 | 5.00 |
|  | SD |  | .141 | .000 | .294 | .000 | .000 |
|  | Median |  | 5.00 | 5.00 | 5.00 | 5.00 | 5.00 |
|  | Min |  | 4 | 5 | 4 | 5 | 5 |
|  | Max |  | 5 | 5 | 5 | 5 | 5 |
|  | IQR |  | 0 | # | 0 | # | # |
| CLEAR Relevance score | Mean | ChatGPT | 4.97 | 4.94 | 5.00 | 4.96 | 4.97 |
|  | SD |  | .172 | .250 | .000 | .196 | .169 |
|  | Median |  | 5.00 | 5.00 | 5.00 | 5.00 | 5.00 |
|  | Min |  | 4 | 4 | 5 | 4 | 4 |
|  | Max |  | 5 | 5 | 5 | 5 | 5 |
|  | IQR |  | 0 | 0 | # | 0 | 0 |
|  | Mean | Gemini | 4.96 | 4.94 | 4.95 | 4.96 | 4.97 |
|  | SD |  | .198 | .250 | .213 | .196 | .169 |
|  | Median |  | 5.00 | 5.00 | 5.00 | 5.00 | 5.00 |
|  | Min |  | 4 | 4 | 4 | 4 | 4 |
|  | Max |  | 5 | 5 | 5 | 5 | 5 |
|  | IQR |  | 1 | 0 | 0 | 0 | 0 |
|  | Mean | DeepSeek | 4.99 | 5.00 | 4.95 | 5.00 | 5.00 |
|  | SD |  | .101 | .000 | .213 | .000 | .000 |
|  | Median |  | 5.00 | 5.00 | 5.00 | 5.00 | 5.00 |
|  | Min |  | 4 | 5 | 4 | 5 | 5 |
|  | Max |  | 5 | 5 | 5 | 5 | 5 |
|  | IQR |  | 0 | # | 0 | # | # |
|  | Mean | Co-Pilot | 4.97 | 5.00 | 4.86 | 5.00 | 5.00 |
|  | SD |  | .172 | .000 | .351 | .000 | .000 |
|  | Median |  | 5.00 | 5.00 | 5.00 | 5.00 | 5.00 |
|  | Min |  | 4 | 5 | 4 | 5 | 5 |
|  | Max |  | 5 | 5 | 5 | 5 | 5 |
|  | IQR |  | 0 | # | 0 | # | # |
| Usefulness score | Mean | ChatGPT | 2.61 | 2.69 | 2.55 | 2.54 | 2.66 |
|  | SD |  | .603 | .704 | .510 | .582 | .639 |
|  | Median |  | 3.00 | 3.00 | 3.00 | 3.00 | 3.00 |
|  | Min |  | 1 | 2 | 2 | 1 | 2 |
|  | Max |  | 4 | 4 | 3 | 3 | 4 |
|  | IQR |  | 1 | 1 | 1 | 1 | 1 |
|  | Mean | Gemini | 2.79 | 2.88 | 2.77 | 2.69 | 2.83 |
|  | SD |  | .540 | .500 | .612 | .549 | .514 |
|  | Median |  | 3.00 | 3.00 | 3.00 | 3.00 | 3.00 |
|  | Min |  | 2 | 2 | 2 | 2 | 2 |
|  | Max |  | 4 | 4 | 4 | 4 | 4 |
|  | IQR |  | 1 | 0 | 1 | 1 | 0 |
|  | Mean | DeepSeek | 3.38 | 3.38 | 3.27 | 3.46 | 3.40 |
|  | SD |  | .509 | .500 | .456 | .582 | .497 |
|  | Median |  | 3.00 | 3.00 | 3.00 | 3.50 | 3.00 |
|  | Min |  | 2 | 3 | 3 | 2 | 3 |
|  | Max |  | 4 | 4 | 4 | 4 | 4 |
|  | IQR |  | 1 | 1 | 1 | 1 | 1 |
|  | Mean | Co-Pilot | 2.78 | 2.56 | 2.91 | 2.69 | 2.86 |
|  | SD |  | .526 | .512 | .526 | .549 | .494 |
|  | Median |  | 3.00 | 3.00 | 3.00 | 3.00 | 3.00 |
|  | Min |  | 2 | 2 | 2 | 2 | 2 |
|  | Max |  | 4 | 3 | 4 | 4 | 4 |
|  | IQR |  | 1 | 1 | 0 | 1 | 0 |
| GQS score | Mean | ChatGPT | 3.97 | 4.19 | 3.95 | 3.65 | 4.11 |
|  | SD |  | .597 | .544 | .653 | .629 | .471 |
|  | Median |  | 4.00 | 4.00 | 4.00 | 4.00 | 4.00 |
|  | Min |  | 2 | 3 | 2 | 2 | 3 |
|  | Max |  | 5 | 5 | 5 | 5 | 5 |
|  | IQR |  | 0 | 1 | 0 | 1 | 0 |
|  | Mean | Gemini | 4.19 | 4.25 | 4.23 | 4.23 | 4.11 |
|  | SD |  | .509 | .577 | .528 | .514 | .471 |
|  | Median |  | 4.00 | 4.00 | 4.00 | 4.00 | 4.00 |
|  | Min |  | 3 | 3 | 3 | 3 | 3 |
|  | Max |  | 5 | 5 | 5 | 5 | 5 |
|  | IQR |  | 0 | 1 | 1 | 1 | 0 |
|  | Mean | DeepSeek | 4.84 | 4.75 | 4.68 | 4.96 | 4.89 |
|  | SD |  | .370 | .447 | .477 | .196 | .323 |
|  | Median |  | 5.00 | 5.00 | 5.00 | 5.00 | 5.00 |
|  | Min |  | 4 | 4 | 4 | 4 | 4 |
|  | Max |  | 5 | 5 | 5 | 5 | 5 |
|  | IQR |  | 0 | 1 | 1 | 0 | 0 |
|  | Mean | Co-Pilot | 4.03 | 4.06 | 4.09 | 3.88 | 4.09 |
|  | SD |  | .415 | .250 | .610 | .326 | .373 |
|  | Median |  | 4.00 | 4.00 | 4.00 | 4.00 | 4.00 |
|  | Min |  | 3 | 4 | 3 | 3 | 3 |
|  | Max |  | 5 | 5 | 5 | 4 | 5 |
|  | IQR |  | 0 | 0 | 0 | 0 | 0 |

^$^Question categories: 1= Biological and surgical aspects, 2= Prosthetic and material science, 3= Clinical outcomes and complications, and 4= Patient centered and economic factors. #= No interquartile range was calculated due to constant factor. CLEAR= Completeness; Lack of false information; Evidence support; Appropriateness; and Relevance reliability tool, GQS= Global Quality Scale score, SD= Standard deviation, Min= Minimum value, Max= Maximum value, IQR= Interquartile range.

**Supplementary Table 2.** Frequencies and percentages of each scale answer for CLEAR individual elements based on AI chatbot and category of questions in this study (n= 99 questions).

| Variable | Scale answer | AI Chatbot | ^$^Question Category | | | | | | | | | |
| --- | --- | --- | --- | --- | --- | --- | --- | --- | --- | --- | --- | --- |
|  |  |  | All (n=99) | | 1 (n=16) | | 2 (n=22) | | 3 (n=26) | | 4 (n=35) | |
|  |  |  | n | % | n | % | n | % | n | % | n | % |
| CLEAR Completeness score | Poor | ChatGPT | 0 | 0 | 0 | 0 | 0 | 0 | 0 | 0 | 0 | 0 |
|  | Fair |  | 2 | 2.0 | 0 | 0 | 1 | 4.5 | 1 | 3.8 | 0 | 0 |
|  | Good |  | 15 | 15.2 | 7 | 43.8 | 3 | 13.6 | 3 | 11.5 | 2 | 5.7 |
|  | Very good |  | 72 | 72.7 | 5 | 31.3 | 17 | 77.3 | 22 | 84.6 | 28 | 80.0 |
|  | Excellent |  | 10 | 10.1 | 4 | 25.0 | 1 | 4.5 | 0 | 0 | 5 | 14.3 |
|  | Poor | Gemini | 0 | 0 | 0 | 0 | 0 | 0 | 0 | 0 | 0 | 0 |
|  | Fair |  | 0 | 0 | 0 | 0 | 0 | 0 | 0 | 0 | 0 | 0 |
|  | Good |  | 5 | 5.1 | 1 | 6.3 | 3 | 13.6 | 0 | 0 | 1 | 2.9 |
|  | Very good |  | 68 | 68.7 | 8 | 50.0 | 15 | 68.2 | 19 | 73.1 | 26 | 74.3 |
|  | Excellent |  | 26 | 26.3 | 7 | 43.8 | 4 | 18.2 | 7 | 26.9 | 8 | 22.9 |
|  | Poor | DeepSeek | 0 | 0 | 0 | 0 | 0 | 0 | 0 | 0 | 0 | 0 |
|  | Fair |  | 0 | 0 | 0 | 0 | 0 | 0 | 0 | 0 | 0 | 0 |
|  | Good |  | 1 | 1.0 | 0 | 0 | 1 | 4.5 | 0 | 0 | 0 | 0 |
|  | Very good |  | 17 | 17.2 | 4 | 25.0 | 5 | 22.7 | 1 | 3.8 | 7 | 20.0 |
|  | Excellent |  | 81 | 81.8 | 12 | 75.0 | 16 | 72.7 | 25 | 96.2 | 28 | 80.0 |
|  | Poor | Co-Pilot | 0 | 0 | 0 | 0 | 0 | 0 | 0 | 0 | 0 | 0 |
|  | Fair |  | 0 | 0 | 0 | 0 | 0 | 0 | 0 | 0 | 0 | 0 |
|  | Good |  | 12 | 12.1 | 4 | 25.0 | 4 | 18.2 | 3 | 11.5 | 1 | 2.9 |
|  | Very good |  | 70 | 70.7 | 11 | 68.8 | 14 | 63.6 | 21 | 80.8 | 24 | 68.6 |
|  | Excellent |  | 17 | 17.2 | 1 | 6.3 | 4 | 18.2 | 2 | 7.7 | 10 | 28.6 |
| CLEAR Lack of False score | Poor | ChatGPT | 0 | 0 | 0 | 0 | 0 | 0 | 0 | 0 | 0 | 0 |
|  | Fair |  | 0 | 0 | 0 | 0 | 0 | 0 | 0 | 0 | 0 | 0 |
|  | Good |  | 3 | 3.0 | 1 | 6.3 | 1 | 4.5 | 1 | 3.8 | 0 | 0 |
|  | Very good |  | 28 | 28.3 | 2 | 12.5 | 7 | 31.8 | 11 | 42.3 | 8 | 22.9 |
|  | Excellent |  | 68 | 68.7 | 13 | 81.3 | 14 | 63.6 | 14 | 53.8 | 27 | 77.1 |
|  | Poor | Gemini | 0 | 0 | 0 | 0 | 0 | 0 | 0 | 0 | 0 | 0 |
|  | Fair |  | 0 | 0 | 0 | 0 | 0 | 0 | 0 | 0 | 0 | 0 |
|  | Good |  | 0 | 0 | 0 | 0 | 0 | 0 | 0 | 0 | 0 | 0 |
|  | Very good |  | 20 | 20.2 | 2 | 12.5 | 6 | 27.3 | 7 | 26.9 | 5 | 14.3 |
|  | Excellent |  | 79 | 79.8 | 14 | 87.5 | 16 | 72.7 | 19 | 73.1 | 30 | 85.7 |
|  | Poor | DeepSeek | 0 | 0 | 0 | 0 | 0 | 0 | 0 | 0 | 0 | 0 |
|  | Fair |  | 0 | 0 | 0 | 0 | 0 | 0 | 0 | 0 | 0 | 0 |
|  | Good |  | 0 | 0 | 0 | 0 | 0 | 0 | 0 | 0 | 0 | 0 |
|  | Very good |  | 8 | 8.1 | 1 | 6.3 | 4 | 18.2 | 3 | 11.5 | 0 | 0 |
|  | Excellent |  | 91 | 91.9 | 15 | 93.8 | 18 | 81.8 | 23 | 88.5 | 35 | 100.0 |
|  | Poor | Co-Pilot | 0 | 0 | 0 | 0 | 0 | 0 | 0 | 0 | 0 | 0 |
|  | Fair |  | 0 | 0 | 0 | 0 | 0 | 0 | 0 | 0 | 0 | 0 |
|  | Good |  | 0 | 0 | 0 | 0 | 0 | 0 | 0 | 0 | 0 | 0 |
|  | Very good |  | 30 | 30.3 | 4 | 25.0 | 7 | 31.8 | 10 | 38.5 | 9 | 25.7 |
|  | Excellent |  | 69 | 69.7 | 12 | 75.0 | 15 | 68.2 | 16 | 61.5 | 26 | 74.3 |
| CLEAR Evidence score | Poor | ChatGPT | 0 | 0 | 0 | 0 | 0 | 0 | 0 | 0 | 0 | 0 |
|  | Fair |  | 0 | 0 | 0 | 0 | 0 | 0 | 0 | 0 | 0 | 0 |
|  | Good |  | 0 | 0 | 0 | 0 | 0 | 0 | 0 | 0 | 0 | 0 |
|  | Very good |  | 6 | 6.1 | 1 | 6.3 | 2 | 9.1 | 2 | 7.7 | 1 | 2.9 |
|  | Excellent |  | 93 | 93.9 | 15 | 93.8 | 20 | 90.9 | 24 | 92.3 | 34 | 97.1 |
|  | Poor | Gemini | 0 | 0 | 0 | 0 | 0 | 0 | 0 | 0 | 0 | 0 |
|  | Fair |  | 0 | 0 | 0 | 0 | 0 | 0 | 0 | 0 | 0 | 0 |
|  | Good |  | 0 | 0 | 0 | 0 | 0 | 0 | 0 | 0 | 0 | 0 |
|  | Very good |  | 8 | 8.1 | 2 | 12.5 | 2 | 9.1 | 2 | 7.7 | 2 | 5.7 |
|  | Excellent |  | 91 | 91.9 | 14 | 87.5 | 20 | 90.9 | 24 | 92.3 | 33 | 94.3 |
|  | Poor | DeepSeek | 0 | 0 | 0 | 0 | 0 | 0 | 0 | 0 | 0 | 0 |
|  | Fair |  | 0 | 0 | 0 | 0 | 0 | 0 | 0 | 0 | 0 | 0 |
|  | Good |  | 0 | 0 | 0 | 0 | 0 | 0 | 0 | 0 | 0 | 0 |
|  | Very good |  | 2 | 2.0 | 0 | 0 | 1 | 4.5 | 1 | 3.8 | 0 | 0 |
|  | Excellent |  | 97 | 98.0 | 16 | 100.0 | 21 | 95.5 | 25 | 96.2 | 35 | 100.0 |
|  | Poor | Co-Pilot | 0 | 0 | 0 | 0 | 0 | 0 | 0 | 0 | 0 | 0 |
|  | Fair |  | 0 | 0 | 0 | 0 | 0 | 0 | 0 | 0 | 0 | 0 |
|  | Good |  | 0 | 0 | 0 | 0 | 0 | 0 | 0 | 0 | 0 | 0 |
|  | Very good |  | 5 | 5.1 | 1 | 6.3 | 3 | 13.6 | 1 | 3.8 | 0 | 0 |
|  | Excellent |  | 94 | 94.9 | 15 | 93.8 | 19 | 86.4 | 25 | 96.2 | 35 | 100.0 |
| CLEAR Appropriateness score | Poor | ChatGPT | 0 | 0 | 0 | 0 | 0 | 0 | 0 | 0 | 0 | 0 |
|  | Fair |  | 0 | 0 | 0 | 0 | 0 | 0 | 0 | 0 | 0 | 0 |
|  | Good |  | 2 | 2.0 | 0 | 0 | 1 | 4.5 | 0 | 0 | 1 | 2.9 |
|  | Very good |  | 12 | 12.1 | 7 | 43.8 | 3 | 13.6 | 1 | 3.8 | 1 | 2.9 |
|  | Excellent |  | 85 | 85.9 | 9 | 56.3 | 18 | 81.8 | 25 | 96.2 | 33 | 94.3 |
|  | Poor | Gemini | 0 | 0 | 0 | 0 | 0 | 0 | 0 | 0 | 0 | 0 |
|  | Fair |  | 0 | 0 | 0 | 0 | 0 | 0 | 0 | 0 | 0 | 0 |
|  | Good |  | 17 | 17.2 | 3 | 18.8 | 7 | 31.8 | 4 | 15.4 | 3 | 8.6 |
|  | Very good |  | 10 | 10.1 | 1 | 6.3 | 0 | 0 | 1 | 3.8 | 8 | 22.9 |
|  | Excellent |  | 72 | 72.7 | 12 | 75.0 | 15 | 68.2 | 21 | 80.8 | 24 | 68.6 |
|  | Poor | DeepSeek | 0 | 0 | 0 | 0 | 0 | 0 | 0 | 0 | 0 | 0 |
|  | Fair |  | 0 | 0 | 0 | 0 | 0 | 0 | 0 | 0 | 0 | 0 |
|  | Good |  | 0 | 0 | 0 | 0 | 0 | 0 | 0 | 0 | 0 | 0 |
|  | Very good |  | 2 | 2.0 | 0 | 0 | 1 | 4.5 | 0 | 0 | 1 | 2.9 |
|  | Excellent |  | 97 | 98.0 | 16 | 100.0 | 21 | 95.5 | 26 | 100.0 | 34 | 97.1 |
|  | Poor | Co-Pilot | 0 | 0 | 0 | 0 | 0 | 0 | 0 | 0 | 0 | 0 |
|  | Fair |  | 0 | 0 | 0 | 0 | 0 | 0 | 0 | 0 | 0 | 0 |
|  | Good |  | 0 | 0 | 0 | 0 | 0 | 0 | 0 | 0 | 0 | 0 |
|  | Very good |  | 2 | 2.0 | 0 | 0 | 2 | 9.1 | 0 | 0 | 0 | 0 |
|  | Excellent |  | 97 | 98.0 | 16 | 100.0 | 20 | 90.9 | 26 | 100.0 | 35 | 100.0 |
| CLEAR Relevance score | Poor | ChatGPT | 0 | 0 | 0 | 0 | 0 | 0 | 0 | 0 | 0 | 0 |
|  | Fair |  | 0 | 0 | 0 | 0 | 0 | 0 | 0 | 0 | 0 | 0 |
|  | Good |  | 0 | 0 | 0 | 0 | 0 | 0 | 0 | 0 | 0 | 0 |
|  | Very good |  | 3 | 3.0 | 1 | 6.3 | 0 | 0 | 1 | 3.8 | 1 | 2.9 |
|  | Excellent |  | 96 | 97.0 | 15 | 93.8 | 22 | 100.0 | 25 | 96.2 | 34 | 97.1 |
|  | Poor | Gemini | 0 | 0 | 0 | 0 | 0 | 0 | 0 | 0 | 0 | 0 |
|  | Fair |  | 0 | 0 | 0 | 0 | 0 | 0 | 0 | 0 | 0 | 0 |
|  | Good |  | 0 | 0 | 0 | 0 | 0 | 0 | 0 | 0 | 0 | 0 |
|  | Very good |  | 4 | 4.0 | 1 | 6.3 | 1 | 4.5 | 1 | 3.8 | 1 | 2.9 |
|  | Excellent |  | 95 | 96.0 | 15 | 93.8 | 21 | 95.5 | 25 | 96.2 | 34 | 97.1 |
|  | Poor | DeepSeek | 0 | 0 | 0 | 0 | 0 | 0 | 0 | 0 | 0 | 0 |
|  | Fair |  | 0 | 0 | 0 | 0 | 0 | 0 | 0 | 0 | 0 | 0 |
|  | Good |  | 0 | 0 | 0 | 0 | 0 | 0 | 0 | 0 | 0 | 0 |
|  | Very good |  | 1 | 1.0 | 0 | 0 | 1 | 4.5 | 0 | 0 | 0 | 0 |
|  | Excellent |  | 98 | 99.0 | 16 | 100.0 | 21 | 95.5 | 26 | 100.0 | 35 | 100.0 |
|  | Poor | Co-Pilot | 0 | 0 | 0 | 0 | 0 | 0 | 0 | 0 | 0 | 0 |
|  | Fair |  | 0 | 0 | 0 | 0 | 0 | 0 | 0 | 0 | 0 | 0 |
|  | Good |  | 0 | 0 | 0 | 0 | 0 | 0 | 0 | 0 | 0 | 0 |
|  | Very good |  | 3 | 3.0 | 0 | 0 | 3 | 13.6 | 0 | 0 | 0 | 0 |
|  | Excellent |  | 96 | 97.0 | 16 | 100.0 | 19 | 86.4 | 26 | 100.0 | 35 | 100.0 |

CLEAR= Completeness; Lack of false information; Evidence support; Appropriateness; and Relevance reliability tool. ^$^Question categories: 1= Biological and surgical aspects, 2= Prosthetic and material science, 3= Clinical outcomes and complications, and 4= Patient centered and economic factors.

Supplementary Table 3. Correlations between the FKG and SMOG scores of the responses in each AI chatbot (n= 99).

| ^$^Factor | Statistic | FKG Vs SMOG scores |
| --- | --- | --- |
| ChatCPT | r | .929 |
|  | P | <.001 |
| Gemini | r | .961 |
|  | P | <.001 |
| DeepSeek | r | .941 |
|  | P | <.001 |
| Co-Pilot | r | .898 |
|  | P | <.001 |

FKG= Flesh-Kinkaid Grade Level, SMOG= Simple Measure of Gobbledygook readability index, r= Pearson’s correlation coefficient, P= Probability value.

Supplementary Table 4. Correlations between the FKG, SMOG, GQS, usefulness, and CLEAR scores of the responses in each AI chatbot (n= 99).

| Factor | Variables | Statistic | GQS scores | Usefulness scores | CLEAR scores |
| --- | --- | --- | --- | --- | --- |
| ChatGPT | FKG scores | Rho | -.051 | -.325 | -.162 |
|  |  | P | .619 | .001 | .109 |
|  | SMOG scores | Rho | -.064 | -.322 | -.183 |
|  |  | P | .530 | .001 | .070 |
|  | GQS scores | Rho | 1.000 | .553 | .659 |
|  |  | P | - | <.001 | <.001 |
|  | Usefulness scores | Rho | .553 | 1.000 | .554 |
|  |  | P | <.001 | - | <.001 |
|  | CLEAR scores | Rho | .659 | .554 | 1.000 |
|  |  | P | <.001 | <.001 | - |
| Gemini | FKG scores | Rho | -.240 | -.178 | -.319 |
|  |  | P | .017 | .077 | .001 |
|  | SMOG scores | Rho | -.207 | -.178 | -.247 |
|  |  | P | .040 | .077 | .014 |
|  | GQS scores | Rho | 1.000 | .467 | .698 |
|  |  | P | - | <.001 | <.001 |
|  | Usefulness scores | Rho | .467 | 1.000 | .615 |
|  |  | P | <.001 | - | <.001 |
|  | CLEAR scores | Rho | .698 | .615 | 1.000 |
|  |  | P | <.001 | <.001 | - |
| DeepSeek | FKG scores | Rho | -.122 | .141 | -.107 |
|  |  | P | .229 | .163 | .293 |
|  | SMOG scores | Rho | -.126 | .119 | -.074 |
|  |  | P | .213 | .241 | .464 |
|  | GQS scores | Rho | 1.000 | .321 | .736 |
|  |  | P | - | .001 | <.001 |
|  | Usefulness scores | Rho | .321 | 1.000 | .417 |
|  |  | P | .001 | - | <.001 |
|  | CLEAR scores | Rho | .736 | .417 | 1.000 |
|  |  | P | <.001 | <.001 | - |
| Co-Pilot | FKG scores | Rho | -.040 | -.080 | -.201 |
|  |  | P | .691 | .431 | .046 |
|  | SMOG scores | Rho | -.024 | -.086 | -.220 |
|  |  | P | .815 | .396 | .028 |
|  | GQS scores | Rho | 1.000 | .479 | .549 |
|  |  | P | - | <.001 | <.001 |
|  | Usefulness scores | Rho | .479 | 1.000 | .520 |
|  |  | P | <.001 | - | <.001 |
|  | CLEAR scores | Rho | .549 | .520 | 1.000 |
|  |  | P | <.001 | <.001 | - |

FKG= Flesh-Kinkaid Grade Level, SMOG= Simple Measure of Gobbledygook readability index, CLEAR= Completeness; Lack of false information; Evidence support; Appropriateness; and Relevance reliability tool, GQS= Global Quality Scale, Rho= Spearman’s correlation coefficient, P= Probability value.
